# Crp Induces Switching of the CsrB and CsrC RNAs in *Yersinia pseudotuberculosis* and Links Nutritional Status to Virulence

**DOI:** 10.3389/fcimb.2012.00158

**Published:** 2012-12-17

**Authors:** Ann Kathrin Heroven, Maike Sest, Fabio Pisano, Matthias Scheb-Wetzel, Rebekka Steinmann, Katja Böhme, Johannes Klein, Richard Münch, Dietmar Schomburg, Petra Dersch

**Affiliations:** ^1^Abteilung Molekulare Infektionsbiologie, Helmholtz-Zentrum für InfektionsforschungBraunschweig, Germany; ^2^Institut für Bioinformatik und Biochemie, Technische Universität BraunschweigBraunschweig, Germany; ^3^Institut für Mikrobiologie, Technische Universität BraunschweigBraunschweig, Germany

**Keywords:** *Yersinia*, metabolism, gene regulation, virulence, *crp*, *csrA*

## Abstract

Colonization of the intestinal tract and dissemination into deeper tissues by the enteric pathogen *Yersinia pseudotuberculosis* demands expression of a special set of virulence factors important for the initiation and the persistence of the infection. In this study we demonstrate that many virulence-associated functions are coregulated with the carbohydrate metabolism. This link is mediated by the carbon storage regulator (Csr) system, including the regulatory RNAs CsrB and CsrC, and the cAMP receptor protein (Crp), which both control virulence gene expression in response to the nutrient composition of the medium. Here, we show that Crp regulates the synthesis of both Csr RNAs in an opposite manner. A loss of the *crp* gene resulted in a strong upregulation of CsrB synthesis, whereas CsrC levels were strongly reduced leading to downregulation of the virulence regulator RovA. Switching of the Csr RNA involves Crp-mediated repression of the response regulator UvrY which activates *csrB* transcription. To elucidate the regulatory links between virulence and carbon metabolism, we performed comparative metabolome, transcriptome, and phenotypic microarray analyses and found that Crp promotes oxidative catabolism of many different carbon sources, whereas fermentative patterns of metabolism are favored when *crp* is deleted. Mouse infection experiments further demonstrated that Crp is pivotal for a successful *Y. pseudotuberculosis* infection. In summary, placement of the Csr system and important virulence factors under control of Crp enables this pathogen to link its nutritional status to virulence in order to optimize biological fitness and infection efficiency through the infectious life cycle.

## Introduction

The enteric pathogen *Yersinia pseudotuberculosis* is able to survive and proliferate in a variety of environmental reservoirs (e.g., soil, plants, and insects) as well as warm-blooded animals (livestock, wild animals, and birds). In humans *Y. pseudotuberculosis* initiates a large variety of gut-associated diseases (yersiniosis), including enterocolitis, diarrhea, and mesenterial lymphadenitis and is transmitted by the fecal oral route (Bottone, 1999; Koornhof et al., 1999). This lifestyle requires the bacterium to coordinate the expression of physiological traits and virulence determinants to persist in environmental and host niches and promote pathogenesis. In particular the colonization of the intestinal tract of each host affords a precise adjustment of their adhesion/invasion factors, immune defense systems and metabolic adaptation to the nutrient fluxes of their environment (Titgemeyer and Hillen, 2002). To accomplish this task, *Y. pseudotuberculosis* has evolved sophisticated sensory and regulatory mechanisms, which allow the bacteria to sense and react to abrupt and pronounced changes of the carbon source composition. This response permits the preferred utilization of the most efficiently metabolizable carbohydrates in the host niches for optimal biological fitness, and is often used to control various steps of the infection process (Poncet et al., 2009).

**Figure 1:**
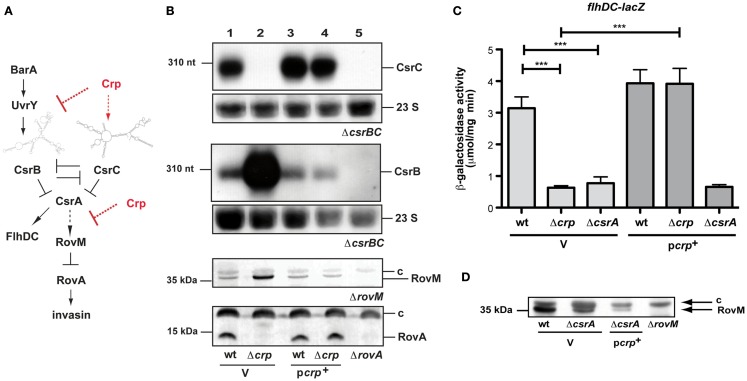
**cAMP receptor protein influence on the regulatory network of early-stage virulence genes of *Y. pseudotuberculosis***. **(A)** The carbon storage regulator (Csr) system is composed of the RNA-binding protein CsrA and the two regulatory RNAs CsrB and CsrC. Expression and activity of the system components are tightly autoregulated and controlled by the two-component system BarA/UvrY. Upregulation of one or both of the regulatory RNAs lead to the sequestration of CsrA, whereby RovM synthesis is repressed and allows production of RovA, the activator of *inv* transcription. Arrows display direct activation of gene expression or protein synthesis, where as dashed arrows label indirect regulation. T represents repression or inactivation. **(B)** Expression of the CsrB and CsrC RNA, RovM, and RovA in the presence and absence of Crp. Whole cell extracts from overnight cultures of *Y. pseudotuberculosis* wildtype strain YPIII and the mutant strain YP89 (Δ*crp*) harboring the empty vector pAKH85 (V) or the *crp*-encoding plasmid pAKH37 (p*crp*^+^) grown at 25°C were prepared, and analyzed by northern blotting with a CsrC- or CsrB-specific probe (two upper panels), or by western blotting with polyclonal antibodies directed against RovM or RovA (two lower panels). The 23S rRNAs are shown as RNA loading control, the sizes of RNA or protein markers are given on the left. The respective negative controls are loaded in the last lanes: YP79 (Δ*csrBC*, two upper panels), YP3 (Δ*rovA*) thrid panel, YP72 (Δ*rovM*) lowest panel. **(C)** To investigate Crp-dependent *flhDC* expression in the absence of *csrA*, the empty vector pAKH85 (V) or its *crp*^+^ derivative pAKH37 (p*crp*^+^) were transformed into *Y. pseudotuberculosis* strains YPIII, YP89 (Δ*crp*) or YP53 (Δ*csrA*) harboring a *flhDC’-’lacZ* fusion (pAKH58). The bacteria were grown overnight in LB medium at 25°C. β-Galactosidase activity from overnight cultures was determined and is given in μmol min^−1^ mg^−1^ for comparison. The data represent the average ±SD from at least three different experiments each done in duplicate. **(D)** Analysis of Crp-dependent RovM protein levels in the absence of *csrA*. Whole cell extracts from overnight cultures of the *Y. pseudotuberculosis* wildtype strain YPIII and YP53 (Δ*csrA*) harboring the empty vector pAKH85 (V) or the *crp*-encoding plasmid pAKH37 (p*crp*^+^) grown at 25°C were prepared, and analyzed by western blotting with polyclonal antibodies directed against RovM. The negative control YP72 (Δ*rovM*) is loaded in the last lane. The loading controls for the western blots are marked with c.

Coordination of metabolic pathways with pathogenicity mechanisms can be achieved by global regulator systems that govern complex networks and cascades of sensory and regulatory elements in a concerted manner. The carbon storage regulator (Csr) system constitutes an important global post-transcriptional regulator system. It controls translation and stability of multiple target mRNAs implicated in metabolic functions, stress adaptation, and virulence in *Yersinia* and many other important pathogens (Timmermans and Van Melderen, 2010; Heroven et al., 2012). The *Yersinia* Csr system consists of (i) the RNA-binding protein CsrA, which usually binds in the Shine–Dalgarno region of target mRNAs, represses translation initiation, and leads to mRNA destabilization (Romeo et al., 2012), and (ii) two small Csr-type regulatory RNAs, CsrB, and CsrC, which are able to bind and inactivate multiple CsrA molecules (Heroven et al., 2008). Sequestration of CsrA by the Csr RNAs is crucial for the initiation of a *Y. pseudotuberculosis* infection, since free CsrA inhibits the expression of the major virulence regulator RovA of *Y. pseudotuberculosis* – the activator of the primary cell entry factor invasin (InvA; Nagel et al., 2001; Heroven et al., 2004, 2008; Tran et al., 2005). CsrA-mediated repression of RovA synthesis occurs indirectly via the LysR-type regulator protein RovM shown to bind within the *rovA* regulatory region and inhibit *rovA* transcription (Heroven and Dersch, 2006; Figure 1A).

CsrA-mediated control of the RovM-RovA-InvA virulence cascade is strongly affected by changes in carbon source availability through alterations of the Csr RNAs, in particular CsrC (Nagel et al., 2001; Heroven and Dersch, 2006; Heroven et al., 2008). CsrC is highly expressed in complex media with high amino acid/peptide concentrations, but it is repressed in media with glucose as single or predominant carbon source (Nagel et al., 2001; Heroven et al., 2008). This suggested a link between the virulence cascade and the cAMP/cAMP receptor protein (Crp) carbon catabolite repression (CCR) system in *Yersinia*.

The Crp is a crucial global regulator that controls the transcription of multiple genes and operons in bacteria of the *Enterobacteriaceae* family, dependent on the supply of glucose or other efficiently utilizable sugars (Saier, 1998; Zheng et al., 2004). Crp is activated by binding cAMP. The cAMP-Crp complex controls expression of its target genes through interaction with a conserved Crp box consensus sequence (TGTGA-N_6_-TCACA) located within their promoter regions (Gunasekera et al., 1992; Busby and Ebright, 1999). Presence of glucose leads to a strong reduction of cAMP and Crp levels which prevents the bacteria from catabolizing alternative sugars, a process called CCR (Ishizuka et al., 1994).

In *Y. pestis*, loss of Crp was found to affect transcription of at least 6% of the genes. Among them are many genes involved in the metabolism of carbon sources and virulence. The latter include the targeted Yop effector proteins YpkA and YopJ, and the plasminogen activator Pla, a virulence factor important for bubonic and pneumonic plague (Kim et al., 2007; Zhan et al., 2008, 2009). As a consequence, the disruption of *crp* led to a >15.000-fold loss in virulence of *Y. pestis* after subcutaneous infection (Zhan et al., 2008). Absence of Crp was also shown to affect *Y. enterocolitica* virulence after oral infection. Examination of gene expression revealed that genes encoding components of the Ysc, Ysa, and the flagellar type III secretion systems (TTSSs) are targets of the cAMP-Crp complex (Petersen and Young, 2002). All these studies indicate that the observed attenuation might reflect altered functioning of the TTSSs crucial for the immune defense during pathogenesis.

In the present work, we disclosed that Crp also regulates the RovM-RovA-InvA regulatory cascade via the Csr system in *Y. pseudotuberculosis* important for the very early-stages of the infection process. Data shown here demonstrate that loss of *crp* strongly affects the levels of the regulatory RNAs CsrC and CsrB which results in a strong upregulation of RovM and repression of RovA and invasin. The comparative analysis of metabolic and transcriptional changes and substantial difference in virulence between *Y. pseudotuberculosis* and an isogenic *crp* mutant further suggests that the cAMP-Crp regulatory system is tightly intertwined with the Csr system and plays an important role connecting carbohydrate metabolism and *Yersinia* virulence.

## Results and Discussion

### Crp controls rovA through the CsrABC-RovM signaling cascade

**Figure A1:**
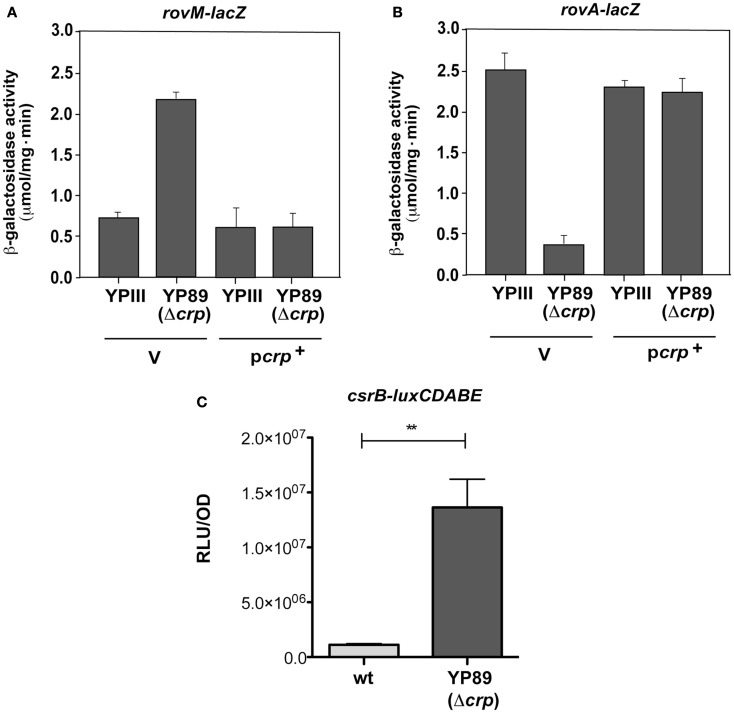
**Analysis of *rovM* and *rovA* transcription in the presence and absence of Crp in *Y. pseudotuberculosis***. The empty vector pAKH85 (V) or its *crp*^+^ derivative pAKH37 (p*crp*^+^) were transformed into *Y. pseudotuberculosis* YPIII and YP89 (Δ*crp*) harboring a *rovM-lacZ* fusion (pAKH63) **(A)** or a *rovA-lacZ* fusion (pAKH47) **(B)**. The bacteria were grown overnight in LB medium at 25°C. β-Galactosidase activity from overnight cultures was determined and is given in μmol min^−1^ mg^−1^ for comparison. The data represent the average ±SD from at least three different experiments each done in duplicate. **(C)** *Y. pseudotuberculosis* strains YPIII and YP89 (Δ*crp*) harboring a *csrB-luxCDABE* reporter fusion (pWO25) were diluted 1:50 in fresh LB medium from overnight cultures and grown for 12 h to early stationary phase at 25°C. Bioluminescence was determined as described in Section “Materials and Methods” and is given as relative light units (RLU) per optical density at 600 nm (OD).

In order to test whether early-stage virulence genes (e.g., invasin) important for the initiation of a *Y. pseudotuberculosis* infection are influenced by the cAMP/Crp system and whether the Csr and Crp systems are linked to control carbohydrate metabolism and virulence, we deleted the *crp* gene and tested expression of each regulator of the *CsrABC-RovM-RovA* cascade. Loss of Crp caused a significant change in the levels of the regulatory RNAs CsrB and CsrC (Figure 1B), but did not alter the amount of CsrA (data not shown). In agreement with previous results (Heroven et al., 2008), the CsrC RNA is highly expressed in *Y. pseudotuberculosis* YPIII at 25°C during stationary phase, conditions that allow optimal expression of the virulence regulator RovA due to reduced expression of RovM. However, in the absence of Crp, CsrC is not detectable. In contrast, the CsrB RNA, which is only weakly expressed in the wildtype, is highly upregulated. Switch of CsrB and CsrC levels is accompanied by a strong induction of RovM production, resulting in full inhibition of RovA synthesis (Figure 1B). The same regulation pattern was obtained when expression of the *rovM* and *rovA* gene was examined by *lacZ* indicator fusions (Figure A1 in Appendix). Expression of *crp* from a plasmid-encoded copy fully restored production of the regulatory factors (Figure 1B), indicating that the changes in regulator levels are solely due to the loss of Crp.

It is well known that Crp affects genes required for virulence, growth on different carbon sources, and survival under carbon, nitrogen, and phosphate limitations in several pathogens (Reverchon et al., 1997; Skorupski and Taylor, 1997; Kennedy et al., 1999; Petersen and Young, 2002). However, only for *Salmonella enterica* serovar Typhimurium it was reported that the Csr system is under the influence of catabolite repression/cAMP-Crp. In contrast to *Yersinia*, both regulatory RNAs and *csrA* were activated by cAMP/Crp in this pathogen (Teplitski et al., 2003, 2006), indicating that the organization of the regulatory network varies between both pathogens.

### Crp activates csrC and represses csrB transcription

**Figure 2:**
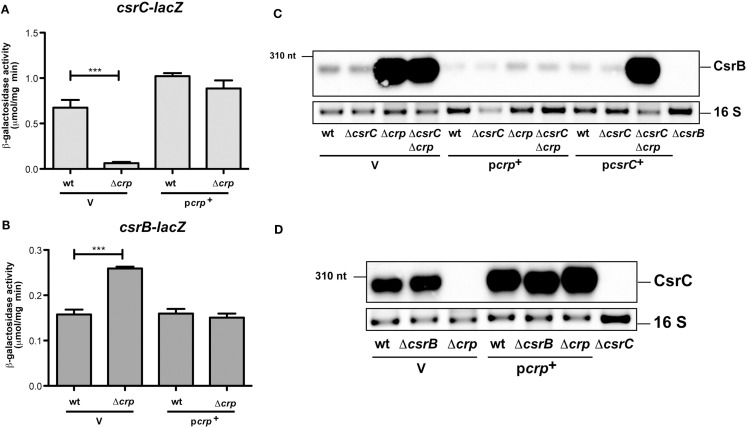
**Analysis of CsrB and CsrC synthesis in the presence and absence of Crp in *Y. pseudotuberculosis***. The empty vector pAKH85 (V) or its *crp*^+^ derivative pAKH37 (p*crp*^+^) were transformed into *Y. pseudotuberculosis* YPIII and YP89 (Δ*crp*) harboring a *csrC-lacZ* fusion (pAKH125) **(A)** or a *csrB-lacZ* fusion (pAKH101) **(B)**. The bacteria were grown overnight in LB medium at 25°C. β-Galactosidase activity from overnight cultures was determined and is given in μmol min^−1^ mg^−1^ for comparison. The data represent the average ±SD from at least three different experiments each done in duplicate. **(C)** Expression of CsrB in the presence and absence of Crp and/or CsrC. Total RNA from of *Y. pseudotuberculosis* wildtype strain YPIII and the mutant strains YP89 (Δ*crp*), YP126 (Δ*csrC*), and YP127 (Δ*crp*Δ*csrC*) harboring the empty vector pAKH85 (V) or the *crp*-encoding plasmid pAKH37 or *csrC*-encoding plasmid pAKH59 grown at 25°C were prepared, and analyzed by northern blotting with a CsrB-specific probe. YP69 (Δ*csrB*) was used as negative control. **(D)** Analysis of CsrC production upon overexpression of Crp in the presence or absence of *csrB*. Total RNA from cultures of *Y. pseudotuberculosis* wildtype strain YPIII and the mutant strains YP89 (Δ*crp*), YP69 (Δ*csrB*), and YP89 (Δ*crp*) harboring the empty vector pAKH85 (V) or the *crp*-encoding plasmid pAKH37 grown at 25°C was prepared, and analyzed by northern blotting with a CsrC-specific probe. YP126 (Δ*csrC*) was used as negative control.

Altered expression pattern of the regulatory cascade suggested that Crp activates expression of the CsrC RNA and/or repression of CsrB synthesis. To examine this possibility, *csrB-lacZ* and *csrC-lacZ* fusions were examined and tested in the wildtype and the *crp* mutant. As shown in Figures 2A,B both reporter fusions are differentially regulated in the Δ*crp* strain. Transcription of the *csrC-lacZ* fusion is drastically reduced, whereas *csrB-lacZ* expression is increased. This phenotype can be complemented, indicating that switch of Csr RNA expression is controlled on the transcriptional level. To confirm Crp-mediated activation of *csrB* transcription we also used a *csrB-luxCDABE* reporter fusion, and found that expression of this reporter was also significantly increased (Figure A1C in Appendix).

**Figure A2:**
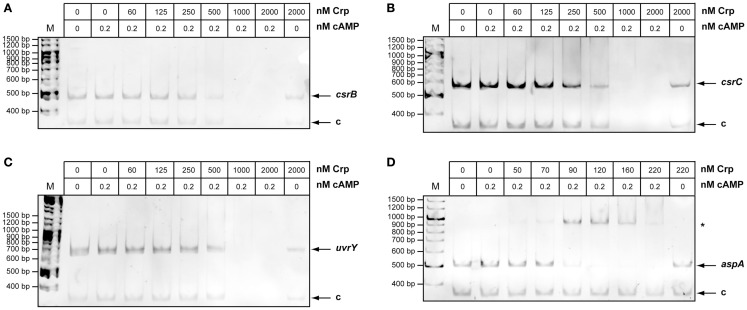
**Interaction of Crp with the *csrB*, *csrC*, *uvrY*, and *aspA* regulatory regions**. The DNA promoter fragments were incubated with increasing concentrations of Crp and 0.2 mM cAMP. A sample with the highest protein concentration without cAMP was used a snegative control (right lane). The Crp-DNA complexes were separated on a 4% polyacrylamide gel. The position of specific higher molecular weight complexes is marked with an asterisk. A molecular weight standard M was loaded, and the corresponding molecular weights are indicated on the left. The analyzed fragments were the following: *csrB* (−399 to +5) **(A)**, *csrC* (−500 to +4) **(B)**, *uvrY* (−507 to +6 relative to translational start) **(C)**, and *aspA* (−422 to +22 relative to translational start) **(D)**.

Expression of the Csr RNAs is autoregulated in a way that (over)production of one RNA leads to a reduced expression of the other, and vice versa (Figure 1A; Heroven et al., 2008). Thus, Crp-mediated switching of the Csr RNA synthesis could occur by activation of *csrC*, thereby repressing expression of *csrB* via the autoregulatory mechanism. To investigate this possibility we first analysed the influence of Crp on CsrB levels in the absence of *csrC*. Upregulation of the CsrB RNA in the absence of Crp was also detectable in a *csrC/crp* double mutant, suggesting that strong induction of CsrB synthesis does not primarily occur through downregulation of *csrC* (Figure 2C). This result was confirmed by data, demonstrating that CsrB increase in the Δ*crp*Δ*csrC* strain can be complemented by Crp, but an overexpression of CsrC had no negative effect on CsrB levels. Since overexpression of Crp resulted in an increase in CsrC levels (Figures 1B and 2D), we further tested whether this induction is still detectable in the absence of CsrB. In fact, higher levels of CsrC were identified in the wildtype (Figure 2D, lane 4) and the *csrB* mutant (Figure 2D, lane 5), indicating that repression of *csrC* in a *crp* mutant cannot be entirely the result of the increased expression of *csrB* in the *crp* deletion strain. Taken together, the Crp-induced switch of Csr RNA expression seems to occur mainly through upregulation of CsrB, but *csrC* expression is also further induced by Crp in an CsrB-independent manner (Figure 1A). We further overexpressed and purified the Crp protein and studied the interaction of the regulator with *csrB* and *csrC* promoter fragments in the presence of cAMP, but no specific binding of Crp to both regulatory regions was detectable. However, a specific nucleoprotein complex was formed with the *aspA* promoter fragment (Figure A2 in Appendix) shown to bind Crp in *Y. pestis* (Zhan et al., 2008). This suggested that Crp-mediated regulation of *csrB* and *csrC* expression is indirect.

An interesting aspect in this context is the observation that a switch from CsrC to CsrB in the *crp* mutant has very strong downstream consequences on the Csr regulatory cascade (Figure 1B). We previously showed that similar to the expression of CsrC, also expression of CsrB antagonizes the activity of CsrA and leads to a strong downregulation of *rovM* and activation of *rovA* upon induction (Heroven et al., 2008). Why upregulation of CsrB in the absence of Crp is insufficient to fulfill this function is unclear. One possibility is that Crp, in addition to controlling the BarA/UvrY and Csr regulons, controls additional regulatory factors of the RovM-RovA-InvA virulence cascade downstream of CsrA. In this case, overexpression of CsrB would lead to the sequestration of CsrA, and this would affect expression of other CsrA targets in the *crp*-deficient mutant. To test this hypothesis, we analyzed expression of an *flhDC’-‘lacZ* fusion. CsrA was previously shown to bind and stabilize the *flhDC* transcript, and reduction of CsrA levels was found to reduce *flhDC’-‘lacZ* expression (Figure 1C; Heroven et al., 2008). Expression of the reporter fusion was also significantly decreased, indicating that the amount of functional CsrA is reduced in the *crp*-deficient strain, and overexpression of Crp was not able to restore *flhDC* expression in the *csrA* mutant (Figure 1C). In addition, we found that Crp is still able to reduce the level of the RovM protein in the absence of CsrA (Figure 1D, lanes 2 and 3). This strongly indicates that Crp also affects expression of the *rovM-rovA-invA* regulatory cascade downstream of the Csr system (Figure 1A).

### Influence of Crp on Hfq and the BarA/UvrY system

**Figure A3:**
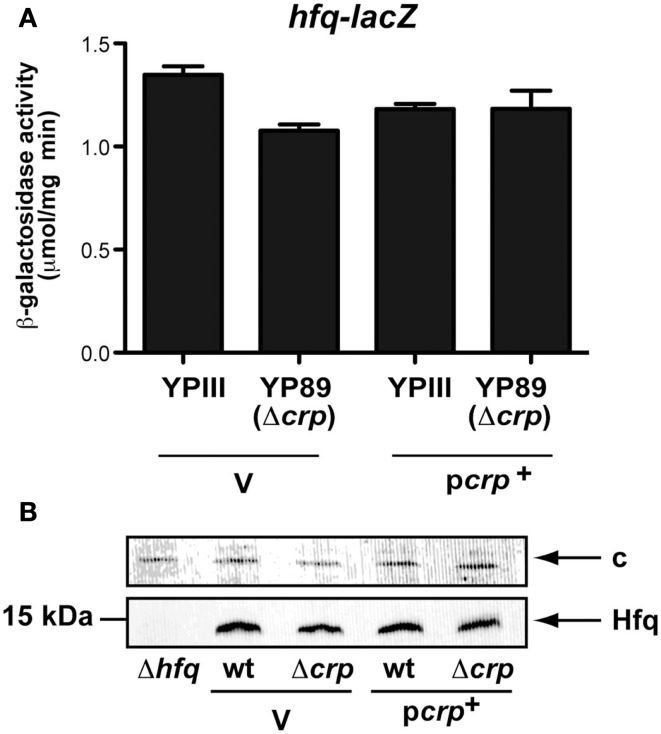
**Analysis of *hfq* transcription in the presence and absence of Crp in *Y. pseudotuberculosis***. **(A)** The empty vector pAKH85 (V) or its *crp*^+^ derivative pAKH37 (p*crp*^+^) were transformed into *Y. pseudotuberculosis* YPIII and YP89 (Δ*crp*) harboring an *hfq-lacZ* fusion (pAKH105). The bacteria were grown overnight in LB medium at 25°C. β-Galactosidase activity from overnight cultures was determined and is given in μmol min^−1^ mg^−1^ for comparison. The data represent the average ±SD from at least three different experiments each done in duplicate. **(B)** Expression of Hfq in the presence and absence of Crp. Whole cell extracts from overnight cultures of *Y. pseudotuberculosis* wildtype strain YPIII and the mutant strains YP89 (Δ*crp*) harboring the empty vector pAKH85 (V) or the *crp*-encoding plasmid pAKH37 (p*crp*^+^) grown at 25°C were prepared, and analyzed by western blotting with polyclonal antibodies directed against Hfq. The negative control YP80 (Δ*hfq*) is loaded in the last lane. The loading control for the western blot is marked with c.

The hexameric RNA chaperone Hfq, which is important for proper folding and stability of many regulatory RNAs, has previously been shown to increase the abundance of Csr-type RNAs (Sonnleitner et al., 2006; Sorger-Domenigg et al., 2007). Based on this result, we first examined whether the Crp-mediated effect on the abundance of the Csr RNAs can be explained by an alteration of *hfq* expression. However, no major effect on the transcription of an *hfq-lacZ* fusion and intracellular Hfq levels was observed when *crp* was absent (Figure A3 in Appendix).

**Figure 3:**
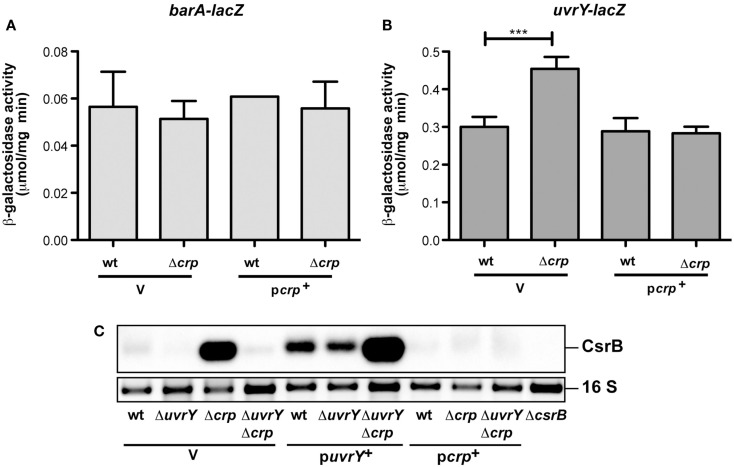
**Analysis of *barA* and *uvrY* transcription in the presence and absence of Crp in *Y. pseudotuberculosis***. The empty vector pAKH85 (V) or its *crp*^+^ derivative pAKJH37 (p*crp*^+^) were transformed into *Y. pseudotuberculosis* YPIII and YP89 (Δ*crp*) harboring a *barA-lacZ* fusion (pKB6) **(A)** or an *uvrY-lacZ* fusion (pKB7) **(B)**. The bacteria were grown overnight in LB medium at 25°C. β-Galactosidase activity from overnight cultures was determined and is given in μmol min^−1^ mg^−1^ for comparison. The data represent the average ±SD from at least three different experiments each done in duplicate. **(C)** Expression of CsrB in the presence and absence of UvrY and/or Crp. Total RNA of *Y. pseudotuberculosis* wildtype strain YPIII and the mutant strains YP89 (Δ*crp*), YP120 (Δ*uvrY*), and YP128 (Δ*crp*Δ*uvrY*) harboring the empty vector pAKH85 or the *uvrY*-encoding plasmid pAKH75 or *crp*-encoding plasmid pAKH37 grown at 25°C was prepared, and analyzed by northern blotting with a CsrB-specific probe. YP69 (Δ*csrB*) was used as negative control.

In a previous work we demonstrated that transcription of *csrB*, but not *csrC* is activated by the BarA/UvrY two-component system composed of the BarA sensor kinase and its cognate response regulator UvrY (Heroven et al., 2008). This is different to homologous systems, such as BarA/UvrY in *Escherichia coli*, BarA/SirA of *S. enterica*, GacS/GacA of *Pseudomonas aeruginosa*, and VarS/VarA of *Vibrio cholerae*. They activate all known Csr-type RNAs in these microorganisms (Heroven et al., 2008, 2012). The *barA/uvrY* system is only weakly expressed in *Y. pseudotuberculosis* under *in vitro* growth conditions in accordance with low CsrB levels, but *csrB* transcription can be induced by overexpression of UvrY (Heroven et al., 2008). Based on this result, we tested whether CsrB upregulation in the *crp* mutants is due to the induction of the *barA* or the *uvrY* gene. Expression of a *barA-lacZ* fusion was not changed in the absence of Crp (Figure 3A), yet a slight and significant increase was detectable for *uvrY-lacZ* expression (Figure 3B). We further assessed CsrB production by northern blotting in a Δ*crp*Δ*uvrY* background to exclude UvrY-mediated activation of *csrB* transcription. CsrB upregulation in the absence of Crp was completely abolished when the *uvrY* gene was absent (Figure 3C, lane 4), but it could be regained by introduction of an UvrY-expressing plasmid (Figure 3C, lane 7). This strongly indicated that Crp-mediated repression of *csrB* occurs through UvrY. However, no specific interaction of the cAMP/Crp complex was detectable (Figure A2 in Appendix), suggesting that Crp influence on *uvrY* expression is indirect. An increase of the CsrB level by the presence of the *uvrY*^+^ plasmid was also detectable in the wildtype and the *uvrY* mutant (Figure 3C, lanes 5 and 6). Yet, the overall amount of CsrB was still lower compared to the Δ*crp*Δ*uvrY* mutant strain (Figure 3C, lanes 5 and 7), indicating that Crp also interferes with the activation of UvrY. Expression of *csrB* and *csrC* in *Salmonella* is also affected by *crp* in a manner that requires the ortholog of UvrY (known as SirA). However, in contrary to the *Yersinia* system, *sirA* is activated by Crp leading to higher CsrB and CsrC levels (Teplitski et al., 2006).

In a previous study we showed that expression of CsrB and CsrC in *Y. pseudotuberculosis* is strongly influenced by the nutrient composition of the medium (Heroven et al., 2008). This suggested that expression of the BarA/UvrY- and Csr-dependent virulence genes might be linked to the metabolic state of the pathogen. The environmental conditions and the physiological signal(s) to which the BarA/UvrY system responds in *Yersinia* are still unknown. It is possible that it senses metabolites that are only present in certain external/host niches. In support of this assumption, expression of *uvrY* was only detectable in *Y. pestis* located in the lung of infected mice, but not in the spleen and liver (Liu et al., 2009). Alternatively, the physiological stimulus might only be produced when certain metabolic functions are active, which are repressed by Crp during growth in LB. In fact, it was reported that metabolic end products such as formate and acetate are able to induce the BarA sensor kinase in *E. coli* (Chavez et al., 2010), and another finding exists in which an imbalance of the TCA cycle was shown to influence GacS(BarA) signaling in pseudomonads (Takeuchi et al., 2009).

### Influence of Crp on *Y. pseudotuberculosis* metabolism

**Figure 4:**
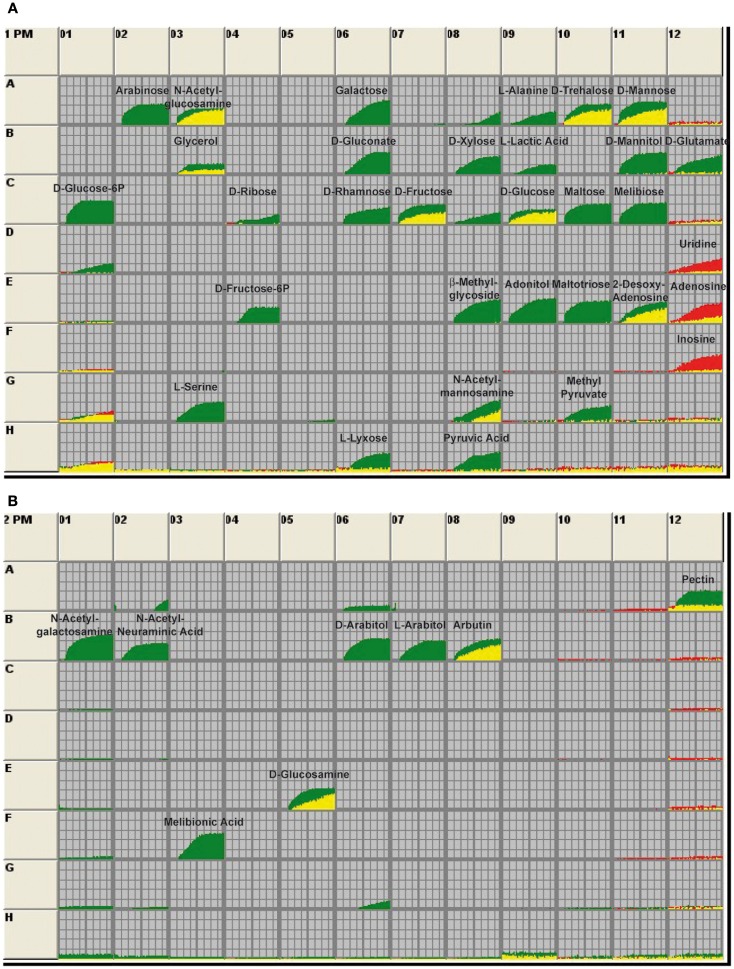
**Utilization of carbon sources by *Y. pseudotuberculosis* wildtype and the *crp* mutant**. The 96-well BIOLOG plates PM1 **(A)** and PM2 **(B)** contain a minimal medium with trace elements except that the C-source is omitted. The various wells in these panels provide 190 different carbon sources. The assays are initiated with the addition of bacterial culture. After incubation, respiration of the bacteria leads to the reduction of a tetrazolium dye, indicating that the bacteria are metabolically active. Information about the carbon sources supplied in the wells is given on the manufacturer’s home page (http://www.biolog.com/pdf/pm_lit/PM1-PM10.pdf). Utilization of the metabolites by the wildtype strain YPIII is given by the green areas in the growth curves and use of the carbon sources by the *crp* mutant YP89 is indicated by the red areas of the growth curves. The data represent one experiment of three independent analyses.

Our present data suggest that the Crp regulon controls metabolic or regulatory factors that hinder CsrB production, but favors expression of CsrC and early-stage virulence genes. Thus far, not much is known about the coordinated adjustment of the metabolism and virulence in *Yersinia*. Since the cAMP/Crp system is mainly responsible for the control of the uptake and utilization of carbon sources, we first tested the ability of the wildtype and the *crp* mutant to metabolize certain carbon sources by the Phenotype MicroArray Technology (Biolog), employing cell respiration as a universal reporter (Bochner et al., 2001; Bochner, 2003). Each well of the array is designed to test utilization of a certain carbon source, and a tetrazolium dye is reduced when the substance is used and the bacteria respire. This reaction is monitored and recorded as green tracing for the wildtype and as red tracing for the *crp* mutant strain. No signal implies that the strains cannot utilize the carbon source (Figure 4). In total, 190 different carbon and energy sources were analysed of which 44 could be readily used by *Y. pseudotuberculosis* when grown at 25°C. A large number of sugars could only be used by the wildtype, but not in the *crp* mutant (e.g., arabinose, galactose, glucose-6-P, fructose-6-P, maltose, mannitol, melibiose, xylose, and arabitol), indicating that expression of the uptake and catabolic functions are part of the CCR process. Crp-dependent utilization of non-glucose substrates seems to be conserved between *Y. pseudotuberculosis* and *Y. pestis*, since *Y. pestis* fermentation of maltose, galactose, and mannitol was also abolished in a *crp* and a *cyaA* mutant (Kim et al., 2007). Some carbon sources could still be used by the *crp* deletion mutant (e.g., d-mannose, d-trehalose, d-fructose, and d-glucose). Most interestingly, other carbon sources, such as the nucleosides uridine, adenosine, and inosine could only be used by the *crp* mutant, but not by the *Y. pseudotuberculosis* wildtype strain (Figure 4A).

**Figure 5:**
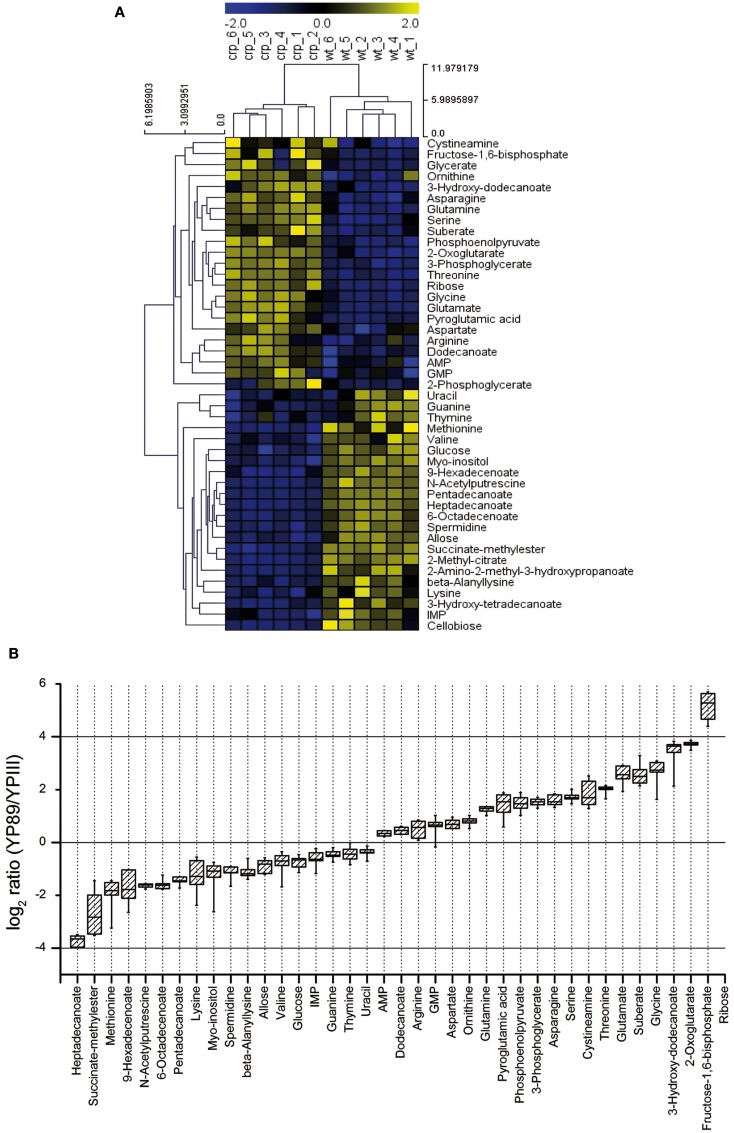
**Metabolic profiles of *Y. pseudotuberculosis* and the *crp* mutant**. **(A)** The metabolite profile was determined from the *Y. pseudotuberculosis* wildtype strain YPIII and the isogenic Δ*crp* strain YP89 (six biological replicates for each strain). Unbiased hierarchical clustering was performed of 45 identified metabolites that were significantly changed in the mutant compared to wildtype (Student’s *t*-test, *p*-value < 0.05). Clustering was performed using average linkage and Euclidean distance. The analysis is visualized by a heatmap in which each square represents the normalized intensity of a metabolite. **(B)** Box-and-whisker plots illustrating metabolites that were significantly changed between the *crp* mutant and the wildtype grown in LB at 25°C.

In order to define metabolic differences between wildtype and the *crp* mutant we also conducted a metabolic profiling approach using GC/MS (for details, see Materials and Methods). One hundred twelve metabolites were detected in the cell extracts (six biological replicates for each strain), with a mean relative standard error of 12.9 and 15.56% for the *crp* mutant and the wildtype, respectively. Seventy six metabolites could be positively identified of which 45 were significantly altered (*p*-value < 0.05) in the *crp* mutant strain (Table S1 in Supplementary Material). An unbiased clustering of the different samples (shown on the *x*-axis) illustrated in the heat map in Figure 5A revealed a metabolite profile for the individual *crp* mutant replicates that clustered together and differed significantly from those of the wildtype (shown on the *y*-axis).

**Figure A4:**
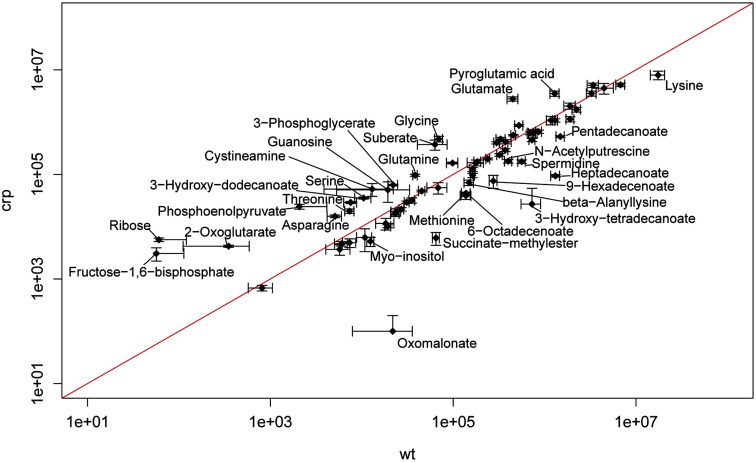
**Scatterplot of the identified 45 metabolites that were significantly changed between the wildtype and the *crp* mutant**. Metabolites which differed ≥2-fold between the wildtype and the *crp* mutant were indicated by names.

A scatterplot illustrates the alteration of multiple metabolites between the *Y. pseudotuberculosis* wildtype strain YPIII and the isogenic *crp* mutant (Figure A4 in Appendix). The most pronounced changes were detected for metabolites related to the central carbon metabolism (glycolysis, TCA cycle), the fatty acid, and the amino acid metabolism. In particular, levels of multiple fatty acids (e.g., heptadecanoate, 9-hexadecenoate, 6-octadecenoate, pentadecanoate) were significantly reduced in the *crp* mutant, whereas 3-hydroxydodecanoate was highly induced (Table S1 in Supplementary Material; Figure 5B). Another effect is the accumulation of various amino acids in the *crp* mutant strain. Levels of glycine, threonine, serine, glutamate, glutamine, and asparagine were significantly increased in the *crp*-deficient strain. This might possibly indicate an upregulation of protein degradation processes. In addition, ribose concentration and levels of several glycolytic metabolites (i.e., phosphoenolpyruvate, 3-phosphoglycerate, and fructose-1,6-bisphosphate) and are drastically increased (Table S1 in Supplementary Material; Figure 5B). Consistent with this, 2-phosphoglycerate and glycerate could only be detected in the *crp* mutant. Furthermore, signifycantly higher amounts of 2-oxoglutarate were detectable in the absence of Crp. Taken together, the changes observed on the metabolic level of the central metabolism, specifically the decrease of fatty acids and increase of amino acids, are in line with a general energy mobilization strategy.

### Transcriptomic changes in comparison of the wildtype and the crp mutant

**Figure 6:**
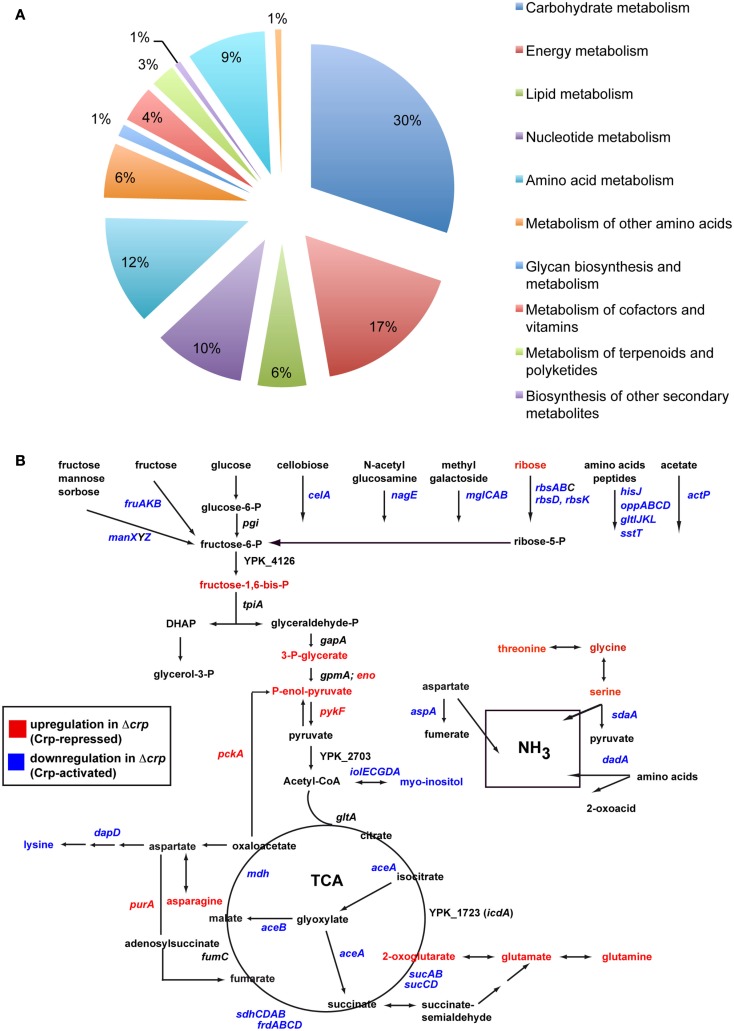
**Global influence of Crp on gene expression and metabolism of *Y. pseudotuberculosis***. **(A)** Differentially regulated genes between the wildtype and the *crp* mutant. Sixteen independent cultures of strains YPIII and YP89 were grown in LB medium at 25°C to early stationary phase, total RNA was isolated and four samples were pooled for RNA-labeling with Cy5 (wildtype) and Cy3 (Δ*crp*). The mixed RNA probes were hybridized to an Agilent microarray carrying 4172 chromosomal and 92 virulence plasmid encoded genes of *Y. pseudotuberculosis*. After normalization, changes in gene expression are given as median values for at least three probes (Table S1 in Supplementary Material). Genes showing overall fold-changes ≥1.8 are included in the displayed diagram of gene classes. **(B)** Central carbon metabolism in *Y. pseudotuberculosis*. Significant alterations in the metabolic and transcriptomic pattern between the wildtype and the *crp* mutant are indicated. An increase of a metabolite in the *crp* mutant is indicated by showing the metabolite in red (Crp-repressed), whereas a decreased metabolite is displayed in blue (Crp-activated). Accordingly, upregulated genes in the *crp* mutant are indicated in red (Crp-repressed), downregulated genes are given in blue (Crp-activated).

The strong influence of Crp on the utilization of carbon sources, the metabolome, and expression of crucial virulence regulators indicate a complex network that adjusts metabolic functions and virulence factor expression to the different conditions encountered by the pathogen during the course of infection. To provide a more comprehensive insight, we wanted to compare changes of the metabolite composition with alterations of the gene expression pattern. To identify the genes under control of Crp when the early virulence genes are activated through the CsrABC-RovM-RovA signaling cascade, a microarray analysis was performed using total RNA isolated from the same *Y. pseudotuberculosis* YPIII (wt) and YP89 (Δ*crp*) culture used for the isolation of the intracellular metabolites. Three hundred sixty eight genes of all 4172 protein-encoding chromosomal genes and of the 92 genes of the *Y. pseudotuberculosis* virulence plasmid varied ≥1.8-fold or more between the wildtype and the *crp* mutant strain. One hundred ninety genes (52%) were upregulated and 178 (48%) down-regulated in the *crp* mutant. Classification according to the genome annotation of *Y. pseudotuberculosis* YPIII showed that altered genes belonged to several functional categories: virulence factors/regulators, metabolism (in particular carbon and energy metabolism), and environmental/stress adaptation (Figure 6A; Table S2 in Supplementary Material). To validate these results, qRT-PCR was performed for 10 identified Crp targets using 5S rRNA as a control, and significant different transcript levels of these target genes were identified in the *crp* mutant strain (Table S3 in Supplementary Material).

#### Pathogenicity

Numerous Crp-dependent transcripts encode virulence-associated traits. Both the primary invasion factor invasin (*invA*) and its transcriptional regulator (*rovA*) became downregulated in the absence of Crp, whereas the LysR-type repressor of the *rovA* gene RovM was upregulated. In addition, the afimbrial adhesin operon (*psaABC*) and the positive activators (*psaEF*) previously shown to be activated by RovA in *Y. enterocolitica* and *Y. pestis* (Cathelyn et al., 2006, 2007), and the *flhDC* operon which is activated by CsrA (Heroven et al., 2008) were downregulated in the *crp* mutant. The *flhDC* genes control the synthesis of flagella and motility which are important for host cell invasion (Young et al., 2000). In contrast, transcript levels of the response regulator *uvrY* were significantly increased when *crp* was deleted. This all together is in full agreement with findings established earlier in this study and confirms that Crp controls the expression of the early virulence gene cascade CsrABC-RovM-RovA-InvA via the response regulator UvrY (Figures 1 and 3). Furthermore, transcripts of the virulence plasmid (pYV) encoding components of the TTSS (e.g., *yscM/lcrQ*, *yscC*, *yopD*, *yopH*, *virG*) which are only weakly expressed under the applied growth conditions are further repressed in the *crp* negative strain. Previous studies demonstrated that a *crp* mutation reduced Yop secretion in *Y. enterocolitica* and *Y. pestis* grown at 37°C under calcium-depleted conditions (Petersen and Young, 2002; Kim et al., 2007). Thus, Crp seems to act as a positive regulator of Yop secretion in all three pathogenic yersiniae. In addition, many other virulence-associated genes were found to be controlled by Crp, among them are the urease genes important to create a more favorable less acidic microenvironment during transfer through the stomach (Burne and Chen, 2000), genes for autoinducer synthesis, iron acquisition, host cell interaction, and manipulation (Table S2 in Supplementary Material).

#### Environmental/stress adaptation

Many Crp-dependent genes are involved in the adaptation of the bacteria to environmental changes and are important for the biological fitness within host tissues. The *pspG* gene of the phage shock protein response system, which is required for virulence when the Ysc TTSS is induced (Darwin and Miller, 2001), is downregulated in the absence of Crp. In addition, cold shock (*cspC*, *cspD*), carbon starvation (*cstA*, *dps*), general stress (*uspA*), and acid resistance genes (*hdeBD*) were repressed in the *crp* mutant, whereas other stress-associated genes (e.g., *sodC*) and several protease genes (*hflCXK*, *rseP*, *opdA/prlC*, *htrA*) were upregulated. The increase in the amino acid levels in the metabolic profile of the *crp* mutant (Table S2 in Supplementary Material) could be, at least in part, a result of increased protein degradation. Notably, the serin protease HtrA was also shown to facilitate resistance of *Yersinia* to oxidative stress and promotes survival in macrophages (Williams et al., 2000).

#### Metabolism

Various enzymes involved in the TCA cycle (*rcs*, *mdh*, *sdhCDAB*, *frdABCD*, *sucABCD*), the glyoxylate bypass, and acetate utilization (*iclR*, *aceBA*, *fdoG*), underwent downregulation in the absence of Crp. Repression of the TCA cycle genes is reflected in the elevation of 2-oxoglutarate levels (Figures 5 and 6B; Table S1 in Supplementary Material). Cytochromes and ATP synthase subunits (*cyoAB*, *atpIFE*) were also repressed indicating that oxidative phosphorylation is reduced in the absence of *crp*. In addition, several phosphotransferase systems, permeases and ABC transporter, and enzymes (e.g., *fruAKB*, *fruD*, *manXYZ*, *rbsAB*, *rbsD*, *rbsK*, *mglBAC*) that facilitate uptake, phosphorylation, and catabolism of carbohydrates were decreased in the expression level. In contrast, glycolytic enzymes (*glgAP*, *eno*, *pckA*, *rcs*, *pykF*) were strongly repressed in the presence of Crp (Figure 6B; Table S2 in Supplementary Material). It is likely that altered levels of the phosphonolpyruvate, 3-phosphoglycerate, and fructose-1,6-bisphosphate (Figure 5; Table S1 in Supplementary Material) are the result of these alterations. Taken together, Crp seems to induce oxidative phosphorylation mediated by the TCA and promotes the ability to utilize a variety of different carbohydrates.

Many genes involved in the amino acid catabolism were also strongly reduced in the *crp* mutant (Table S2 in Supplementary Material). Some of these enzymes account for the release of metabolic ammonia via reactions that promote deamination of amino acids and formation of α-keto acids entering the TCA cycle which may account for the strong increase in threonine, glycine, and serine levels (Table S1 in Supplementary Material). Downregulation of genes involved in fatty acid uptake and metabolism explains the significant decrease of multiple fatty acids in the *crp* mutant (Figure 5; Table S1 in Supplementary Material). Several genes of the nucleotide catabolism (e.g., *nupC1*) were also upregulated in the *crp* mutant strain (Table S2 in Supplementary Material) which could be involved in the increased utilization of nucleosides in the phenotyping array. Crp was further shown to repress *relA*. RelA mediates the stringent response to amino acid starvation and deprivation of other energy sources by catalyzing the synthesis of ppGpp, which interacts with the RNA polymerase and alters the transcription profile. A recent study revealed that ppGpp and CsrA regulate numerous genes in *E. coli* in a reciprocal fashion (Edwards et al., 2011). ppGpp induces CsrB and CsrC synthesis antagonizing CsrA activity. CsrA post-transcriptionally represses RelA and thereby ppGpp synthesis (Edwards et al., 2011). Crp-mediated changes of the Csr RNA levels, leading to an overall reduction of the CsrA activity (Figures 1 and 2), would interfere with this feedback loop and would result in an increase of RelA and ppGpp and a decrease in the components of the translational and the transcription machinery (Barker et al., 2001a,b). In fact, a large number of genes affecting genetic information processing, including those encoding ribosomal proteins, tRNA synthases, sigma factors, a RNA helicase, and elongation factors were upregulated in the absence of Crp (Table S2 in Supplementary Material). In *E. coli*, also protein degradation is influenced by an increase of ppGpp levels (Kuroda et al., 2001). A similar scenario is likely for the *Yersinia* *crp* mutant in which multiple amino acids are accumulated (Table S1 in Supplementary Material). Taken together, our data indicate that Crp of *Y. pseudotuberculosis* is a key regulator that controls the Csr system and connected metabolic, virulence, and stress functions.

### The Crp protein affects colonization of mesenterial lymph nodes and organs

**Figure 7:**
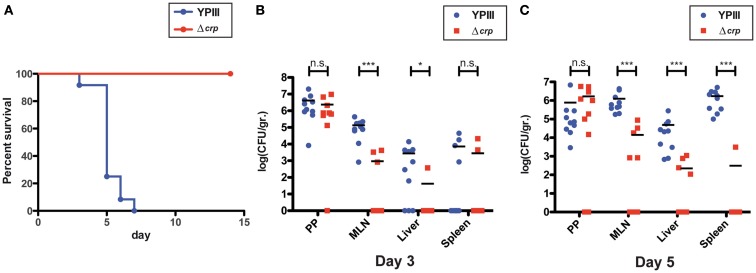
**Influence of *crp* on the survival of mice orally infected with *Y. pseudotuberculosis***. **(A)** Survival of BALB/c mice (*n* = 12/strain) was monitored after oral infection with 5·10^8^ CFU of *Y. pseudotuberculosis* YPIII (blue) and of the *crp* mutant YP88 (red) up to 14 days. **(B,C)** Influence of *crp* on the colonization of the lymphatic tissues and organs in mice orally infected with *Y. pseudotuberculosis*. BALB/c mice were infected intragastrically with an inoculum of 10^8^ CFU of *Y. pseudotuberculosis* wildtype YPIII or the *crp* mutant. After 3 **(B)** and 5 days **(C)** of infection, mice were sacrificed and the number of bacteria in homogenized host tissues and organs was determined by plating. Data are represented in scatter plots of numbers of CFU per gram as determined by counts of viable bacteria on plates. The statistical significances between the wildtype and the *crp* mutant were determined by the Mann–Whitney-test. *P*-values: *<0.05; ***<0.001.

The large variety of Crp-dependent virulence-linked genes suggested that Crp plays a crucial role for *Y. pseudotuberculosis* pathogenesis by adjusting virulence genes expression according to the nutrient conditions encountered during the course of the infection. To examine influence of Crp on virulence, we used the mouse model for infection and compared survival and colonization of host tissues by the *Y. pseudotuberculosis* wildtype strain (YPIII) and the isogenic *crp* mutant (YP89). First, groups of BALB/c mice (*n* = 12) were infected orally with approximately 5·10^8^ bacteria. Their survival was followed over 2 weeks and date of death was recorded (Figure 7A). Only mice infected with wildtype bacteria showed signs of infection (e.g., weight loss) and died of the infection between days 4 and 6, whereas mice infected with the *crp*-deficient strain did not develop disease symptoms and were still alive 14 days after infection.

To complement the survival assay, we quantified the number of bacteria present in the Peyer’s patches (PPs), the mesenterial lymph nodes (MLNs), liver, and spleen 3 and 5 days post infection with 10^8^ bacteria. Most interestingly, similar numbers of the wildtype and the mutant strain were present in the PP at day 3 and 5, indicating that loss of *crp* has no effect on the colonization and survival in these lymphoid nodules of the small intestine (Figure 7B). In contrast, significantly reduced numbers (100-fold less) of the *crp* mutant were recovered from the MLNs, and an even more pronounced effect was observed in the liver and spleen of the infected mice after 5 days of infection (Figure 7C). These results clearly indicated that presence of Crp is advantageous for the colonization and/or persistence in the MLNs, and essential for the colonization of the organs later during the infection. How deregulation of the CsrA-RovM-RovA-InvA virulence cascade contributes to this phenotype needs to be elucidated. However, influence on early-stage virulence factors seems less important, as the most severe effect on pathogenesis was observed during later stage of the infection.

A similar dramatic attenuation was observed for *Y. pestis*, in particular by the subcutaneous route of infection, indicating that Crp is crucial for the development of both bubonic plague and gastrointestinal diseases caused by its closest relative. In both bacteria, Crp seems crucial for dissemination from early infection sites (peripheral lymph/PPs) to deeper tissues. Strong reduction of virulence has been attributed to the loss of Pla, type III secretion, and effector protein expression (Zhan et al., 2009), but changes of many additional virulence factors and alteration of the metabolic activity and stress/environmental adaptation certainly contribute to the dramatic loss of virulence. The Crp protein is also important for the virulence of *Y. enterocolitica* (Petersen and Young, 2002). However, Crp-mediated control of early-stage virulence factors and effect on pathogenesis appear to be somewhat different. Invasion and colonization of the PPs and the MLNs by *Y. enterocolitica* and the *crp* mutant variant was similar up to 3 days post infection, but was decreased 6 days post infection. Crp influence on certain virulence factors might vary between *Y. enterocolitica* and *Y. pseudotuberculosis*. In fact, in contrast to *Y. pseudotuberculosis* expression of invasin and urease was not affected in a *Y. enterocolitica* *crp* mutant (Petersen and Young, 2002).

## Conclusion

Many virulence factors of pathogenic yersiniae are controlled by nutrients. Coupling of virulence gene expression with metabolic adaptation during the course of an infection does not only allow to save energy for uptake and utilization of the most efficient carbon and energy sources, it also ensures that only required virulence factors are expressed to support colonization and survival of the host immune response. Previous work clearly demonstrated that the global regulatory systems cAMP/Crp and the Csr system, which are involved in the control of the carbon and energy metabolism, modulate expression of a subset of virulence functions. Both systems are crucial to achieve a fine-tuned response of the bacterium to changes of the host environment. In this study we present evidence that the Crp and Csr regulons are tightly connected in *Yersinia*.

The Crp protein controls the level of both regulatory Csr RNAs, whereby expression of CsrB is strongly upregulated and CsrC is fully repressed in the absence of the *crp* gene. This switch of Csr RNA expression is mostly achieved through UvrY-mediated activation of *csrB* transcription. However, a modest CsrB-independent activation of *csrC* via Crp was also observed. Consistent with the proposed regulatory hierarchy (Figure 1A), there is a considerable overlap between the regulons controlled by Crp and CsrA (Table S2 in Supplementary Material). The switch between the regulatory RNAs is accompanied by a drastic change of the primary metabolism. In particular, uptake, catabolism, and terminal oxidation of alternative carbon sources (carbohydrates, fatty acids, amino acids) is inhibited, while glycolysis and macromolecular synthesis (proteins, nucleotides, glycogen, and lipids) is favored.

Several direct targets of Crp have recently been identified in *Y. pestis* (Zhan et al., 2008), and with a few exceptions (e.g., *gntT*, *idnO*, *idnK* gluconate transport, and metabolism), all of them have also been found to be Crp-dependent in *Y. pseudotuberculosis*. Numerous genes have also been demonstrated to be Crp-dependent in *Salmonella* and *E. coli*, indicating that the Crp regulon is considerably conserved among enteropathogens. Intriguingly, the majority of Crp-activated genes implicated in the TCA cycle, glycolysis, lipid, nitrogen/amino acid, and nucleotide metabolism were also activated by an upshift in growth temperature from 26 to 37°C, whereas the majority of Crp-repressed genes is also repressed at 37°C (Han et al., 2004; Motin et al., 2004). This suggests that reorganization of the metabolic pathways to accommodate to different life styles (environmental reservoirs/flea vector – mammalian host) in response to temperature might involve Crp.

How switching of the Csr-type RNAs and the imbalance of the metabolic functions are connected remains elusive. Initial microarray experiments indicate that CsrA of *Yersinia* controls multiple metabolic pathways besides the RovM-RovA-InvA virulence cascade (Heroven et al., 2012; Table S2 in Supplementary Material). This includes enzymes of the glycolytic pathway, gluconeogenesis, amino acid pathways, and TCA cycle, as well as many transport and stress adaptation genes (Ann Kathrin Heroven, unpublished results). In fact, many of them are also controlled by Crp (Table S2 in Supplementary Material), suggesting that these pathways are controlled by Crp through the Csr system. However, it is also possible that metabolic changes (i.e., alteration of certain metabolites) due to the loss of Crp affect the expression and or activity of the BarA/UvrY system, which in turn influence transcription of CsrB and CsrC. A recent study showed that an imbalance of the Krebs cycle (i.e., mutations in the fumerase isoenzym gene *fumA*) can strongly affect expression of the Csr-type RNAs RsmY and RsmZ of *P. fluorescens* in a GacA (UvrY)-dependent manner. An analysis of the intracellular metabolites revealed a strong correlation between Csr RNA expression and the pools of 2-oxoglutarate, succinate, and fumarate (Takeuchi et al., 2009). The concentrations of 2-oxoglutarate was also dramatically altered in the *Yersinia* *crp* mutant. In *Legionella pneumophila*, synthesis of the alarmone ppGpp, which is triggered upon carbon source depletion via the enzymes RelA and SpoT, had an influence on the activity of the GacA/UvrY homologous system LetS/LetA (Molofsky and Swanson, 2003). A similar scenario is possible for UvrY activation in the *Yersinia crp* mutant in which the *relA* gene is upregulated.

In conclusion, our study demonstrates that a tight link exists between Crp and UvrY/Csr-dependent control of the primary metabolism which allows the pathogen to coordinate and fine-tune expression of pathogenicity factors with the nutritional status. Crp is important to stimulate crucial virulence factors (e.g., Yop effectors, adhesins/invasins) as well as other virulence-associated traits (iron uptake, motility, acid stress adaptation), and favors expression of oxidative catabolism of a large variety of carbon sources during proliferation in the mammalian host. Thus, it is not astonishing that *crp* mutants of *Y. pseudotuberculosis* are avirulent. Nonetheless, *Yersinia* *crp* mutants are still able to multiply in host tissues and are as such highly attractive as live vaccine strains. Recent studies demonstrated that an engineered *Y. pestis* strain, in which the amount of Crp becomes depleted upon tissue invasion, is attenuated, immunogenic, and protective against pneumonic plague (Sun et al., 2010, 2011). Based on the fact that Crp is well conserved and crucial for virulence in many relevant Gram-negative bacteria, this protein could also be a suitable target for antimicrobial drug development.

## Materials and Methods

### Cell culture, media, and growth conditions

*Yersinia* strains were routinely grown at 25°C in LB (Luria Bertani) broth. The antibiotics used for bacterial selection were as follows: ampicillin 100 μg ml^−1^, chloramphenicol 30 μg ml^−1^, tetracycline 5 μg ml^−1^, and kanamycin 50 μg ml^−1^.

### Strain and plasmid construction

All DNA manipulations, restriction digestions, ligations, and transformations were performed using standard genetic and molecular techniques as described previously (Miller, 1992; Sambrook, 2001). Plasmids used in this study are listed in Table S4 in Supplementary Material and primers for plasmid generation are listed in Table S5 in Supplementary Material.

For construction of the suicide mutagenesis plasmid pAKH3 the *sacB* gene of pAY01 was subcloned into the *Sal*I site of the suicide vector pGP704. The *crp*^+^ fragment of *Y. pseudotuberculosis* of pAKH37 was generated by PCR using primers 409 and 410, digested with *Bam*HI and *Sal*I, and inserted into pACYC184. To generate pAKH105, the upstream regulatory region of *hfq* was amplified using primers I663 and I664 and cloned into the *Eco*RI site of pGP20. Plasmid pAKH125 carries a PCR generated fragment harboring the *csrC* promoter region from nucleotide −355 (primer II157) to nucleotide +4 (primer I363). The fragment was digested and inserted into the *Sal*I site of pHT124. For construction of the mutagenesis plasmids pAKH149 and pAKH151, a PCR fragment harboring a kanamycin resistance gene inserted into the *csrC* or *uvrY* gene (*csrC*::Kan^R^ or *uvrY*::Kan^R^) was generated. First, the kanamycin resistance gene was amplified using primer pair I661/I662 and plasmid pKD4 as template. Next, the *Yersinia* genomic DNA was used as template to amplify 300–500-bp regions flanking the target genes. The upstream fragment was amplified with a primer pair of which the reverse primer contained additional 20 nt at the 5′-end which were homologous to the start of the kanamycin cassette (*csrC*_up_: primer II850/II851; *uvrY*_up_: primer II858/II859). The downstream fragment was amplified with a primer pair of which the forward primer contained additional 20 nt at the 3′-end which were homologous to the end of the kanamycin cassette (*csrC*_down_: primer II852/II853; *uvrY*_down_: primer II860/II861). In the next step, a PCR reaction was performed with the forward and the reverse primer using the upstream and downstream PCR products of the respective target gene and the *kan* gene fragment as templates. The resulting fragment was digested with *Sac*I and ligated into the *Sac*I site of pAKH3. To monitor *barA* expression, the *barA* promoter region (−550) and 5 nt of the coding region were amplified using primers I350 and I351. The amplified fragment was subsequently inserted into the *Pst*I site of pGP20 resulting in pKB6. For the construction of the translational *uvrY*’-’*lacZ* fusion plasmid pKB7, the *uvrY* promoter region ranging from −559 to +8 nt relative to the translational start site was amplified with primer pair I348/I349 from *Yersinia* chromosomal DNA, digested, and ligated into the *Pst*I site of pGP20. To construct the *csrB*-*luxCDABE* fusion plasmid pWO25, the upstream region of *csrB* ranging from nucleotide −462 to +1 was amplified with primer pair III417 and III418 and inserted into the *Bam*HI and *Sal*I site of pFU98. The *crp* coding sequence of plasmid pAKH171 for overexpression of the N-terminally 6×His-tagged Crp protein was amplified using primer IV787 and IV788. The resulting PCR fragment was inserted into the *Nhe*I and *Xho*I sites of pET28a. All clones were confirmed by sequencing (GATC, Konstanz, Germany, or in-house facility).

The *Y. pseudotuberculosis* mutant strains YP72, YP80, YP88, and YP89 were derived from wildtype strain YPIII. The *csrBC* double mutant YP79 was derived from the *csrB* single mutant YP69. The *Y. pseudotuberculosis* mutant strains were constructed with the RED recombinase system as described previously (Datsenko and Wanner, 2000; Derbise et al., 2003; Heroven et al., 2008). First, the kanamycin resistance gene was amplified using primer pairs I661/I662 and plasmid pKD4 as template (see Table S5 in Supplementary Material). For amplification of the regions flanking the target genes *csrC*, *crp*, *hfq*, or *rovM* primer pairs 636/I659 (*csrC*_up_), I660/639 (*csrC*_down_), II235/II236 (*crp*_up_), II237/II238 (*crp*_down_), I665/I666 (*hfq*_up_), I667/I668 (*hfq*_down_), or I63/I64 (*rovM*) were used. The final PCR fragments containing the kanamycin gene flanked by the up- and downstream regions of the respective target genes were transformed into *Y. pseudotuberculosis* YPIII pKD46 or YP69 pKD46 for construction of the *csrBC* double mutant. Chromosomal integration of the fragment was selected by plating on LB supplemented with kanamycin. The *Yersinia* *crp*::*kan* mutant for the animal experiments was named YP88. The selection of the mutants and removal of the kanamycin resistance gene for generation of YP72, YP79, YP80, and YP89 was performed as described by Datsenko and Wanner (2000).

The *Y. pseudotuberculosis* mutants YP87, YP106, YP124, and YP125 were constructed by homologous recombination using suicide plasmids pAKH149 and pAKH151. Plasmids were mated from S17-1λpir (*tra*^+^) into *Y. pseudotuberculosis* YPIII or the *crp* mutant strain YP89. Transconjugants and mutant strains resulting from excision of the integrated plasmid were selected as described previously (Nagel et al., 2001). Strains harboring the desired phenotype were proven by PCR and sequencing. Removal of the kanamycin resistance gene for generation of YP120, YP126, YP127, and YP128 was performed as described by Datsenko and Wanner (2000).

### RNA isolation and northern detection

Overnight cultures were grown to stationary phase (OD_600_ of 3.5). Two milliliters culture was withdrawn, mixed with 0.2 volume of stop solution (5% water-saturated phenol, 95% ethanol), and snap-frozen in liquid nitrogen. After thawing on ice, bacteria were pelleted by centrifugation (2 min, 14,000 rpm, 4°C), and RNA was isolated using the SV total RNA purification kit (Promega) as described by the manufacturer. RNA concentration and quality were determined by measurement of A_260_ and A_280_. Total cellular RNA (5 μg) was mixed with loading buffer (0.03% bromophenol blue, 4 mM EDTA, 0.1 mg/ml EtBr, 2.7% formaldehyde, 31% formamide, 20% glycerol in 4× MOPS buffer) and was separated on 1× MOPS agarose gels (1.2%), vacuum-blotted onto positively charged membranes (Roche) in 10× SSC and UV cross-linked. Prehybridization, hybridization to DIG-labeled DNA probes and membrane washing were conducted using the DIG luminescent Detection kit (Roche) according to the manufacturer’s instructions. The *csrC* and *csrB* transcripts were detected with a DIG-labeled PCR fragment (DIG-PCR nucleotide mix, Roche) with primer pair 555/556 and I82/583 (Table S5 in Supplementary Material), respectively. All northern blot experiments were performed three times.

### Expression and purification of the *Y. pseudotuberculosis* Crp protein

BL21λDE3 transformed with pAKH171 was grown at 37°C in LB broth to an A_600_ of 0.6. IPTG with a final concentration of 0.2 μM was added to induce the expression of 6His-Crp. The cells were grown for an additional 2 h before being harvested. The pellet was resuspended in lysis buffer (50 mM NaH_2_PO_4_ pH8.0; 300 mM NaCl; 10 mM imidazole; 1 mM MgCl_2_) and the cells were lysed with a French press (120,000 psi). The soluble 6His-Crp extract was separated from insoluble cell material by centrifugation. The 6His-Crp protein was then purified by affinity chromatography on Ni-NTA agarose (Qiagen). The column was washed with four column volumes of washing buffer (50 mM NaH_2_PO_4_ pH8.0; 300 mM NaCl; 50 mM imidazole) and eluted with elution buffer (50 mM NaH_2_PO_4_ pH8.0; 300 mM NaCl; 250 mM imidazole). The purity of the 6His-Crp protein was estimated to be >95%.

### Gel mobility shift assays

For DNA-binding studies the purified 6His-Crp protein was dialyzed against the DNA-binding buffer (20 mM Tris-HCl pH 6.8; 10 mM MgCl_2_; 0.1 mM EDTA; 120 mM KCl; 2 mM M DTT; 5% glycerol; 0.1 mg/ml BSA). The DNA samples (130 μmol PCR fragment) of 400–500 bp upstream region of each gene and a control fragment (−232 to +51 nt relative to the *rovA* translational start) were incubated with increasing amounts of purified 6His-Crp protein in 20 μl binding buffer containing 0.2 mM cAMP for 20 min at 30°C. Samples were mixed with loading dye (Qiagen – GelPilot DNA loading dye, 5×) and then subjected to 4% polyacrylamide gel electrophoresis. The DNA was visualized with ethidiumbromide. The corresponding PCR fragments were synthesized with the following primer pairs: 837/II431 (*uvrY*), II846/I438 (*csrB*), I362/I363 (*csrC*), IV853/IV854 (*aspA*), and 185/147 (*rovA*).

### Gel electrophoresis, preparation of cell extracts, and western blotting

For immunological detection of the Hfq, RovA, and RovM proteins, *Y. pseudotuberculosis* cultures were grown under specific environmental conditions as described. Cell extracts of equal amounts of the bacteria were prepared and separated on a 12 or 15% SDS-PAGE (Sambrook, 2001). Subsequently, the samples were transferred onto an Immobilon-P membrane (Millipore) and probed with a polyclonal antibodies directed against RovA, RovM, and Hfq, as described recently (Heroven and Dersch, 2006). For generation of an Hfq specific antibody, a peptide was synthesized (AKGQSLQDPFLNALRRER) and used for immunization of one rabbit. The resulting Hfq peptide antibody was affinity purified using HPLC (Davids Biotechnologie, Germany). All western blot experiments were performed three times.

### β-Galactosidase and luciferase assays

The activity of the β-galactosidase activity of the *lacZ* fusion constructs was measured in permeabilized cells as described previously (Manoil and Beckwith, 1985; Miller, 1992). The activities were calculated as follows: β-galactosidase activity OD_420_·6.75·OD_600_^−1^·Δt (min)^−1^·Vol (ml)^−1^. Reporter fusions emitting bioluminescence were measured in non-permeabilized cells with a Varioskan Flash (Thermo Scientific) using the SkanIt software (Thermo Scientific) for 1 s per time point. The data are given as relative light units (RLU/OD_600_) from three independent cultures performed in duplicate.

### Quantitative RT-PCR

One-step real-time RT-PCR was performed in triplicate with RNA preparations of six independent cultures using a Rotor-Gene Q thermo cycler (Qiagen). For qRT-PCR analysis RNA was prepared from bacterial cells grown to stationary phase at 25°C using the protocol described for RNA isolation and northern detection. Quantitative RT-PCR was carried out with the SensiFast SYBR No-ROX One-Step Kit (Bioline, Germany) applying the three-step cycling protocol according to the manufacturer. Gene specific-primers used for qRT-PCR amplification are listed in Table S4 in Supplementary Material (*mglB*: IV737/IV738; *udp*: IV741/IV742; *ompX*: IV743/IV744; *nagE*: IV749/IV750; YPK_1818: IV751/IV752; *deaD*: IV753/IV754; *oppA*: IV761/IV762; *dadA*: IV765/IV766; *gltI*: IV767/IV768; YPK_3035: IV769/IV770; 5S RNA: II812/II813) and were designed to produce a 200- to 250-bp amplicon with *Y. pseudotuberculosis* YPIII cDNA as template. The amount of PCR product was quantified by measuring fluorescence of SYBR Green dye. Reported gene expression levels were normalized to levels of the 5S RNA transcript. This gene was used as it exhibited identical expression levels in the wildtype and the *crp* mutant under used experimental conditions. Standard curves were detected during every run for each gene tested and established by comparing transcript levels in serial dilutions of total RNA from a control sample. The relative expression of each gene was calculated as described (Pfaffl, 2001).

### Phenotyping microarray analysis (Biolog)

The *Y. pseudotuberculosis* wildtype strain YPIII and the *crp* mutant YP89 were grown at 25°C on LB plates. Bacteria were swabbed from the plates and suspended in the inoculation fluid IF-0 (Biolog, Hayward, CA, USA) containing 1% (v/v) of the redox dye tetrazolium (Dye Mix A; Biolog, Hayward, CA, USA). Hundred microliters of an 85% transmittance suspension of bacteria were added to each well of the PM plates. Plates were incubated in the OmniLog incubator (Biolog, Hayward, CA, USA) for 72 h with readings taken every 15 min. Through microbial respiration, the tetrazolium dye is reduced to the insoluble violet formazan complex. The data were analyzed with the vendor software OmniLog – PM FM 1.20.02 and OmniLog – PM Par 1.20.02 (Biolog, Hayward, CA, USA). Phenotypes were determined based on the area difference under the kinetic curve of dye formation between the mutant and wildtype, and area differences were mean-centered by plate. Reproducibility of the data was investigated by running multiple replicates of each of the parental and mutant strains on separate days. A significant difference between wildtype and mutant strain was defined as a change >1000 of the integrated kinetic curve area.

### Processing of samples for metabolome analysis

Bacteria were grown at 25°C in LB media until stationary phase and a volume corresponding to 5.3 mg cell dry weight was harvested 2 h after reaching the maximum cell density. The bacteria were separated from the medium by filtration with a three-place manifold (Millipore, Billerica, MA, USA) utilizing polycarbonate filters with a pore-size of 0.22 μm (Isopore, Millipore, Billerica, MA, USA) and washed twice with 0.9% NaCl (w/v) to remove remaining medium components. Six hundred milligrams silica beads (diameter <0,1 μm; Kuhmichel, Ratingen) and three ceramic beads (diameter: 5 mm, Peqlab, Erlangen, Germany) were given to the bacteria and the samples were frozen in liquid nitrogen. Subsequently, the bacteria were resuspended in 0.75 ml cold methanol (−20°C) containing ribitol (*c* = 2 μg/ml) as internal standard for normalization purposes. Subsequently the bacterial cells were disrupted in a Precellys 24 homogenizer (Peqlab, Erlangen, Germany) at −5°C with three cycles at a speed of 6500 rpm for 30 s with breaks of 20 s. About 0.4 ml H_2_O and 0.25 ml chloroform were added and phase separation was obtained by centrifugation (5 min, 15,800 × *g*). About 0.75 ml of the upper polar phase were taken, dried under vacuum at room temperature and stored at −20°C until derivatization. For all metabolome data sets extraction blanks were performed and treated like bacterial cell samples.

### Sample derivatization

At first, samples were dried for 30 min under vacuum prior derivatization. The pellets were derivatized in 40 μl pyridine containing methoxyamine hydrochloride (20 mg/ml) at 30°C for 90 min under constant mixing. After addition of 70 μl *N*-methyl-*N*-trimethylsilyltrifluoroacetamide (MSTFA), samples were incubated for 30 min at 37°C followed by 2 h at 25°C with constant agitation. The samples were centrifuged at 14,000 × *g* for 5 min and the supernatants were used for GC-MS analysis. All samples were analyzed within 24 h after derivatization. A retention index marker (*n*-alcanes ranging from C10…C36 in cyclohexane) was used to convert retention times to retention indices.

### Data acquisition by GC-EI-MS

The samples were also analyzed using the Leco Pegasus 4D GC × GC-TOFMS in GC-TOF mode (Leco Instrumente, Mönchengladbach, Germany) equipped with a MPS 2XL autosampler (Gerstel, Mühlheim a. d. Ruhr, Germany). One microliter of the samples was injected in splitless mode into a cooled injection system (Gerstel, Mühlheim a. d. Ruhr, Germany). After an initial time of 0.2 min at 70°C the temperature was ramped to 280°C at a rate of 12°C/s, followed by an additional constant temperature period at 280°C for 5 min. Gas-chromatography was performed on a 7890 Agilent GC over 35 min on a DB-5MS column (30 mm × 0.25 mm I.D.; J&W Scientific, Folsom, CA, USA). Helium flow was set to constant 1 ml/min. After 1 min at 70°C the temperature was increased to 330°C with 10°C/min followed by an additional constant temperature period at 330°C for 8 min. The transfer line was set to 275°C. Ion source temperature was 250°C. Full scan mass spectra were recorded from *m/z* 45 to 0.600 with 20 scans/s. Data acquisition was done using the ChromaTOF software (version 4.24, Leco).

### Data processing and compound identification

Data analysis was done with Metabolite Detector (version 2.06; Hiller et al., 2009). The software solution supports automatic deconvolution of all mass spectra from a chromatogram and calculates the retention indices based on the retention index marker. In Metabolite Detector chromatograms were analyzed in an untargeted batch analysis. Peaks were identified by comparison of retention index and mass spectrum to a user-defined spectra library using a cut-off of 70% identity. Quantification was done by selected unique fragment ions for each individual metabolite.

### Statistical analysis of the metabolome data and visualization of the data

The metabolome data were subjected to normalization by the internal standard ribitol. An additional normalization step by the mean was done. Afterward simple outlier detection (1,75σ ± median) was performed and the detected outliers were discarded. Peak areas of derivates belonging to one substance were summarized before further data evaluation. Statistical analysis and graphical routines were handled in Excel (Microsoft Cooperation), in R^1^ and the MultiExperiment Viewer (Version 4.8.1)^2^. For calculation of statical significant alteration between the mutant and the respective wildtype samples a two tailed Student’s *t*-test was performed. Hierarchical clustering of metabolite profiles was performed with Euclidean distance and average linkage algorithm. Each square of the heatmap of the intracellular metabolome represents the relative intensity of a metabolite.

### Microarray analysis and data analysis

Sequences used for the design of the microarrays (Agilent, 8 × 15 K format), containing three different 60 nt oligonucleotides for all 4172 chromosomal genes (ORFs >30 codons) of the *Y. pseudotuberculosis* YPIII genome and six probes for the 92 genes of the virulence plasmid pYV of *Y. pseudotuberculosis* strain IP32953, were obtained from the NCBI Genome GenBank (NC_010465 and NC_006153). The ORF-specific oligonucleotides were designed using the webdesign application eArray from Agilent^3^ Sixteen independent cultures of *Y. pseudotuberculosis* YPIII and the *crp* mutant strain YP89 were grown in LB at 25°C. Total RNA was isolated from samples using the SV total RNA purification kit (Promega), and RNA concentration and quality was determined with an Agilent 2100 Bioanalyzer using the RNA Nano 6000 kit as described by the manufacturers. Total RNA of four independent samples was pooled. About 1 μg of the pooled samples was used for RNA-labeling with Cy5 (for wildtype RNA) and Cy3 (for mutant RNA) using the ULS^™^ Fluorescent Labeling Kit for Agilent Arrays (Kreatech). Non-incorporated Cy5/Cy3 was removed by KREA*pure* purification columns as suggested by the manufacturers. The degree of labeling was determined by a Nanodrop (Peqlab). Subsequently, 300 ng Cy5-labeled RNA and 300 ng Cy3-labeled RNA were mixed, fragmented, and hybridized to custom-made Agilent microarray slides (8 × 15 K) using the Agilent gene expression hybridization kit as described by the manufacturer. In general, four biological replicates were employed for each experiment. After washing and drying of the microarray slide, data were scanned using Axon GenePix Personal 4100A scanner and array images were captured using the software package GenePix Pro 6.015.

The processing of the resulting microarray data was done using the software package R^4^ in combination with the “Bioconductor” software framework^5^ (Gentleman et al., 2004). Preprocessing based on the marray package employing the read.GenePix function. Control code for probe selection was adapted to the custom-made Agilent microarray system and a quality control was performed to check for hybridization artifacts and large-scale differences between the microarrays of one experiment. A two-color intensity-dependent normalization (“Lowess” normalization) was applied and if necessary supplemented by scale normalization between different microarrays as described (Yang et al., 2002). Differentially expressed genes were obtained using the limma package and the lmFit function for linear modeling. eBayes was used for significance calculations (Smyth, 2004). The overall fold-changes of a gene represented by at least three probes are given as median values for all probes. The set of resulting differential expressed genes (fold-change ≥1.8) was analyzed employing the topGO package for Gene Ontology (GO) term enrichment (Alexa et al., 2006). MIAME compliant array data were deposited in Gene Expression Omnibus (GEO) database and are available via the following accession number: GSE42206.

### Mouse infections

All animal work was performed in accordance with the European Health Law of the Federation of Laboratory Animal Science Associations (FELASA) with appropriate care and welfare of animals. All efforts were made to minimize suffering of the mice. The protocol was approved by the Niedersächsisches Landesamt für Verbraucherschutz und Lebensmittelsicherheit: animal licensing committee permission no. 33.9.42502-04-055/09.

*Yersinia pseudotuberculosis* YPIII (wildtype) and YP88 (*crp* mutant) were grown overnight at 25°C, washed in sterile PBS and used for intragastrical inoculation of 6–8 weeks old female BALB/c mice (Janvier, France) using a ball-tipped feeding needle. For survival assays, 5·10^8^ bacteria of each strain were applied to different groups of mice, and the survival rate of the mice was determined by monitoring the survival every day for 14 days. To assess the impact of a *crp* deletion in *Y. pseudotuberculosis* on tissue colonization, different groups of BALB/c mice were infected with 10^8^ bacteria of each strain. Three days past infection, mice were sacrificed using CO_2_. PPs, MLNs, liver, and spleen were recovered. Isolated PPs were washed with sterile PBS. Organs were weighed and homogenized for 30 s in sterile PBS using a Polytron PT 2100 homogenizer (Kinematica, Switzerland). Subsequently, they were plated on *Yersinia* selective agar (Oxoid, Germany) in three serial dilutions with or without kanamycin. Colony forming units were determined and are given in cfu per gram organ/tissue. The competitive index in comparison to the wildtype strain YPIII was calculated as described (Monk et al., 2008).

## Conflict of Interest Statement

The authors declare that the research was conducted in the absence of any commercial or financial relationships that could be construed as a potential conflict of interest.

## Supplementary Material

The Supplementary Material for this article can be found online at http://www.frontiersin.org/Cellular_and_Infection_Microbiology/10.3389/fcimb.2012.00158/abstract

Supplementary Table S1 **Changed metabolites between *Y. pseudotuberculosis* YPIII and the *crp* mutant**.

Supplementary Table S2 **Classification of Crp-dependent genes**.

Supplementary Table S3 **qRT-PCR of Crp target transcripts**.

Supplementary Table S4 **Bacterial strains and plasmids**.

Supplementary Table S5 **Oligonucleotides used in this study**.

## Footnotes

^1^https://www.R-project.org

^2^http://www.tm4.org/mev/

^3^http://www.genomics.agilent.com.

^4^http://www.r-project.org

^5^http://www.bioconductor.org
