# Supplementary material for: Crp Induces Switching of the CsrB and CsrC RNAs in Yersinia pseudotuberculosis and Links Nutritional Status to Virulence
Source: Front Cell Infect Microbiol. 2012 Dec 17;2:158. doi: 10.3389/fcimb.2012.00158 (PMC3523269; doi:10.3389/fcimb.2012.00158)
Supplement: Supplementary Table S1 — Changed metabolites between Y. pseudotuberculosis YPIII and the crp mutant. [file 34500_Dersch_DataSheet1.PDF]

Supplementary Material

Table S1: Changed metabolites between *Y. pseudotuberculosis* YPIII and the *crp* mutant

Derivative_Group	Mean crp	Relative standard error[a]	Mean wt	Relative standard error[a]	T-test	Ratio crp/wt
2-Amino-2-methyl-3-hydroxypropanoate	0.00	0.00	9395.79	23.26	0.000625	0.00
2-Methyl-citrate	0.00	0.00	761596.89	8.60	0.000000	0.00
2-Oxoglutarate	4233.14	4.82	349.09	68.15	0.000000	12.13
2-Phosphoglycerate	1215.23	32.30	0.00	0.00	0.004156	NA
3-Hydroxy-dodecanoate	24187.28	10.62	2076.93	0.00	0.000002	11.65
3-Hydroxypyridine	1083938.68	18.03	1170528.62	10.63	0.707452	0.93
3-Hydroxy-tetradecanoate	0	0	728044.47	24.26	0.001389	0
3-Phosphoglycerate	62743.04	3.61	22028.60	12.11	0.000000	2.85
4-Aminobutanoate	4735.44	9.98	5987.33	16.00	0.270414	0.79
5-Methylthio-adenosine	29473.14	4.98	30995.32	6.12	0.539227	0.95
6-Deoxymannose	197809.26	4.07	238797.97	4.92	0.010948	0.83
6-Octadecenoate	43547.92	5.26	138053.82	10.94	0.000011	0.32
9-Hexadecenoate	75011.62	28.12	274490.95	9.71	0.000014	0.27
Adenine	1078231.55	7.05	1301986.50	6.25	0.060168	0.83
Adenosine	31959.90	11.28	35313.47	7.46	0.456014	0.91
Allose	10203.89	6.07	19266.77	7.77	0.000033	0.53
AMP	560577.65	3.08	454905.00	6.16	0.005493	1.23
Arginine	471361.41	8.54	331762.56	11.33	0.020056	1.42
Asparagine	15811.24	6.09	5089.45	17.87	0.000000	3.11
Aspartate	165293.90	4.02	98367.59	14.25	0.000516	1.68
Azelate	673.16	12.37	812.41	29.52	0.604408	0.83
beta-Alanyllysine	69513.90	11.72	149008.01	11.89	0.000878	0.47
Cellobiose	0.00	0.00	38683.22	33.91	0.011307	0.00
Citrate	6226.93	46.49	10800.35	18.76	0.204549	0.58
Cystineamine	52441.94	27.61	12984.52	70.29	0.029669	4.04
Cytosine	622102.01	8.10	696404.81	7.58	0.323623	0.89
Dodecanoate	403745.66	5.22	299360.75	7.11	0.002540	1.35
Dodecanol	3677.94	22.77	5727.63	29.50	0.305641	0.64
Ethanolaminephosphate	124797.01	8.35	162636.94	6.78	0.022593	0.77
Fructose-1,6-bisphosphate	3072.67	28.41	0	0	0.001809	NA
Fumarate	17621.77	11.25	23108.00	10.64	0.102663	0.76
Glcyerophosphoglycerol	19319.00	13.79	23661.24	11.84	0.277778	0.82
Glucose	95569.90	9.70	163074.02	4.84	0.000022	0.59
Glutamate	2772770.47	9.45	448472.78	13.60	0.000000	6.18
Glutamine	96497.55	4.45	38270.41	5.56	0.000000	2.52
Glycerate	1480.10	29.14	0.00	0.00	0.001873	NA
Glycerol-3-phosphate	613193.34	17.88	745919.70	10.89	0.337117	0.82
Glycine	476087.78	12.23	69563.23	9.82	0.000001	6.84
Glycolate	4940.15	18.74	7362.89	16.43	0.133718	0.67
GMP	5076752.10	9.80	3396093.66	14.20	0.025538	1.49
Guanine	236035.07	5.66	321786.79	6.92	0.004523	0.73
Guanosine	50625.71	42.22	19318.66	72.58	0.227607	2.62
Heptadecanoate	93952.23	5.82	1310919.54	11.14	0.000000	0.07
Hexadecenoate	6126.83	97.83	0.00	0.00	0.295866	NA
IMP	457224.79	7.14	728231.35	7.41	0.000497	0.63
Isoleucine	1738102.43	10.43	2229704.06	10.35	0.115124	0.78
Leucine	2037280.19	13.46	1892988.20	14.29	0.712624	1.08
Lysine	7927520.36	18.62	17436121.53	17.69	0.014455	0.45
Malate	21490.25	7.48	27140.48	5.99	0.023369	0.79
Maleate	0.00	0.00	7255.05	55.97	0.105545	0.00
Methionine	39149.65	15.36	137744.84	13.48	0.000112	0.28
Myo-inositol	5238.66	20.18	12443.76	8.95	0.000168	0.42
N-Acetylputrescine	176021.98	7.64	561724.44	9.36	0.000002	0.31
Ornithine	863278.80	4.26	523792.79	10.34	0.000066	1.65
Oxalate	3527183.31	10.93	3261053.22	12.22	0.638021	1.08
Oxomalonate	0	0.00	21810.97	63.78	0.154105	0.00
Pentadecanoate	529686.35	4.15	1484961.73	10.41	0.000013	0.36
Phenylalanine	421601.40	8.90	374951.66	14.92	0.505823	1.12
Phosphate	4451211.33	23.04	4469278.83	26.47	0.990998	1.00
Phosphate-monomethylester	48163.19	3.93	45327.15	8.10	0.513533	1.06
Phosphoenolpyruvate	19878.30	9.54	7354.19	11.52	0.000006	2.70
Putrescine	5168943.20	7.76	6762533.80	11.16	0.086108	0.76
Pyroglutamic acid	3510380.98	11.44	1290021.26	11.42	0.000034	2.72
Pyrophosphate	55663.37	24.60	68438.20	24.79	0.569771	0.81
Pyruvate	11532.96	25.25	18315.58	22.56	0.203585	0.63
Ribonate-1,4-lactone	175113.32	7.43	183878.24	8.41	0.672794	0.95

Ribose	5638.35	7.88	0	0	0.000000	NA
Serine	35376.53	4.36	10474.73	19.95	0.000000	3.38
Spermidine	179157.92	8.25	400608.59	9.14	0.000032	0.45
Suberate	372244.82	21.87	63160.88	35.68	0.001162	5.89
Succinate-methylester	6081.18	27.67	64777.73	8.69	0.000000	0.09
Threonine	29081.46	4.66	7557.18	16.17	0.000000	3.85
Thymine	655185.57	7.37	859667.53	7.32	0.020039	0.76
Tryptophan	167106.31	12.70	178113.68	20.11	0.799475	0.94
Uracil	291409.44	6.11	371561.72	6.61	0.017775	0.78
Valine	1138770.08	11.29	1900036.27	9.81	0.003801	0.60

Data shown are mean values of relative changes and standard deviations calculated from 6 biological replicates of each strain.

[a] relative standard error in %

[b] level of significance ($p < 0.05$) of the difference between the strains tested

Table S2: Classification of Crp-dependent genes

Gene ID	Gene locus	Fold change	Description	Category-Class
**Virulence genes**				
Downregulated loci (Crp-activated)				
pYV0054	*yopD*	-2	effector protein, translocator protein	virulence factor, pYV
pYV0075*	*virG*	-1.7	putative Yop targeting lipoprotein	virulence factor, pYV
pYV0079	*yscC*	-2	type III secretion system, immune defence	virulence factor, pYV
pYV0089	*yscM, lcrQ*	-2	type III secretion system, regulatory protein	virulence factor, pYV
pYV0094	*yopH*	-2	effector protein, protein-tyrosine phosphatase	virulence factor, pYV
**YPK_0280**	*bfd*	-2.5	bacterioferritin-associated ferredoxin	virulence factor
YPK_0792	*yspI; ytbI*	-2	autoinducer synthesis protein	virulence factor
**YPK_1876**	*rovA*	-10	transcriptional regulator	virulence regulation
**YPK_2429**	*invA*	-2.4	invasin, host cell invasion factor	virulence factor
**YPK_2758***	*psaB*	-2.4	pili assembly chaperone	virulence factor
**YPK_2759**	*psaA*	-5	pH6 antigen, protein, colonization factor	virulence factor
**YPK_2760**	*psaF*	-5	regulator of *psaABC* operon	virulence factor
**YPK_2761**	*psaE*	-10	transcriptional regulator	virulence regulation
YPK_3289	*crl*	-2	DNA-binding transcriptional regulator	virulence regulation
Upregulated loci (Crp-repressed)				
YPK_0051		16.3	fimbrial protein	virulence factor
**YPK_0385**	*hcp*	1.9	Hcp1 family type VI secretion system effector	virulence factor
**YPK_1559**	*rovM*	3.8	LysR family transcriptional regulator	transcription
**YPK_1606**	*ompX, ailD*	6.9	Ail-type outer membrane protein, putative virulence factor	virulence factor
**YPK_1761**	*yadE*	9.3	YadA domain-containing protein	virulence factor
YPK_2356	*uvrY*	2	two-component system, NarL family, invasion response regulator	virulence regulation
YPK_2615*	*cnf1*	2	Cytotoxic necrotizing factor	virulence factor
**YPK_3549**		8.3	hypothetical protein, putative type VI secretion protein	virulence factor
**YPK_3550**	*icmF*	3	type VI secretion protein IcmF	virulence factor
**YPK_3551**		4	hypothetical protein, putative type VI secretion protein	virulence factor
**YPK_3552**		5.7	type VI secretion protein	virulence factor
**YPK_3553**		5	putative lipoprotein	virulence factor
**YPK_3554**		5	hypothetical protein, putative type VI secretion protein	virulence factor
**YPK_3555**		5.2	hypothetical protein, putative type VI secretion protein	virulence factor
**YPK_3556**		3.4	pentapeptide repeat-containing protein	membrane and intracellular structural molecules
**YPK_3557**		3.5	pentapeptide repeat-containing protein	membrane and intracellular structural molecules
**YPK_3558**		5.3	type VI secretion system Vgr family protein	virulence factor
**YPK_3559**	*clpV*	3.5	type VI secretion ATPase, *clpB* homolog	virulence factor
**YPK_3560**	*impH*	4.2	type VI secretion protein	virulence factor
**YPK_3561**	*impG*	5.3	type VI secretion protein	virulence factor
**YPK_3562**		9.6	type VI secretion system lysozyme-related protein	virulence factor
**YPK_3563**	*hcp*	3.7	type VI secretion system secreted protein	virulence factor
**YPK_3564**	*impC*	10.2	EvpB family type VI secretion protein	virulence factor
**YPK_3565**		7.4	type VI secretion protein	virulence factor
**YPK_3566**		6.3	ImpA domain-containing protein	virulence factor
**Flagella/motility/chemotaxis**				
Downregulated loci (Crp-activated)				
**YPK_1745**	*flhD*	-5	flagellar transcriptional activator FlhD	cell motility, flagellar
**YPK_1746**	*flhC*	-5	flagellar transcriptional activator FlhC	cell motility, flagellar
**YPK_2381**	*fliC*	-2.5	flagellin	cell motility, flagellar
**Stress adaptation**				
Downregulated loci (Crp-activated)				
**YPK_0120**	*uspA*	-10	universal stress protein A	stress response
YPK_0564	*hdeD*	-3.3	acid-resistance membrane protein	stress response, acid
**YPK_1140**	*hdeB*	-2	acid-resistance protein	stress response, acid
**YPK_1602**	*dps*	-1.7	DNA starvation/stationary phase protection	replication, repair
**YPK_2017**	*cstA*	-2.5	carbon starvation protein CstA	transcription
YPK_2474	*cspC*	-2.5	cold shock-like protein	transcription
YPK_2694	*cspD*	-10	cold-shock DNA-binding domain-containing protein	transcription
YPK_3353	*yfiA*	-2.5	modulation protein of the nitrogen assimilation sigma factor 54 /ribosomal protein	transcription

			0EA	
**YPK_3857**	*pspG*	-2.5	phage shock protein G	stress response
Upregulated loci (Crp-repressed)				
YPK_0116	*opdA/prlC*	2.1	oligopeptidase A	stress response, metabolism
YPK_1073	*rseP*	2.0	zinc metallopeptidase RseP, regulator of sigma E protease	stress, response, metabolism
**YPK_1917**	*hslJ*	2.2	heat-inducible protein	stress response
YPK_3445	*sodC*	1.8	Cu/Zn superoxide dismutase	stress response, oxidative
**YPK_3452**	*htrA*	2.1	serine endoprotease	metabolic/stress adaptation
YPK_3795	*hflC*	1.9	FtsH protease regulator HflC	folding, sorting, degradation
YPK_3796	*hflK*	1.9	FtsH protease regulator HflK	folding, sorting, degradation
YPK_3797	*hflX*	1.9	putative GTPase HflX	folding, sorting, degradation
YPK_3976	*rpoH*	1.8	RNA polymerase heat-shock sigma factor (σ^{32})	transcription, stress response
**Genetic information processing**				
Downregulated loci (Crp-activated)				
YPK_0248	*crp*	-10	cAMP-regulatory protein	transcription
YPK_0354	*hupA*	-2	DNA-binding protein HU-alpha	transcription, metabolism
**YPK_0367**	*iclR*	-2	IclR family transcriptional regulator, acetate operon repressor	transcription, metabolism
YPK_1016	*cadC*	-2.5	transcriptional regulator	transcription
**YPK_1275**	*iscR*	-2	Rrf2 family transcriptional regulator, iron-sulfur cluster assembly transcription factor	transcription
**YPK_1975**	*acoR; prpR*	-2.5	Fis family GAF modulated sigma54 specific transcriptional regulator	transcription
YPK_1996	*araC*	-2.5	AraC family transcriptional regulator, arabinose operon regulatory protein	transcription, metabolism
Upregulated loci (Crp-repressed)				
YPK_0278	*tuf*	2	elongation factor Tu	translation
YPK_0282	*rpsJ*	3	small subunit ribosomal protein S10	translation
YPK_0283	*rplC*	3.2	large subunit ribosomal protein L3	translation
YPK_0284	*rplD*	3.2	large subunit ribosomal protein L4	translation
YPK_0285	*rplW*	3.1	large subunit ribosomal protein L23	translation
YPK_0286	*rplB*	2.8	large subunit ribosomal protein L2	translation
YPK_0287	*rpsS*	2.4	small subunit ribosomal protein S19	translation
YPK_0288	*rplV*	2.8	large subunit ribosomal protein L22	translation
YPK_0289	*rpsC*	2.2	small subunit ribosomal protein S3	translation
YPK_0290	*rplP*	2.2	large subunit ribosomal protein L16	translation
YPK_0291	*rpmC*	2.6	large subunit ribosomal protein L29	translation
YPK_0292	*rpsQ*	2.4	small subunit ribosomal protein S17	translation
YPK_0296*	*rpsN*	1.9	small subunit ribosomal protein S14	translation
YPK_0297*	*rpsH*	1.8	small subunit ribosomal protein S8	translation
YPK_0298	*rplF*	1.9	large subunit ribosomal protein L6	translation
YPK_0299	*rplR*	2.4	large subunit ribosomal protein L18	translation
YPK_0300	*rpsE*	2.1	small subunit ribosomal protein S5	translation
YPK_0301	*rpmD*	2.8	large subunit ribosomal protein L30	translation
YPK_0302	*rplO*	2.6	large subunit ribosomal protein L15	translation
YPK_0304*	*rpmJ*	2.8	large subunit ribosomal protein L36	translation
YPK_0305	*rpsM*	2.8	small subunit ribosomal protein S13	translation
YPK_0306	*rpsK*	2.8	small subunit ribosomal protein S11	translation
YPK_0307	*rpsD*	3	small subunit ribosomal protein S4	translation
YPK_0308	*rpoA*	3.1	DNA-directed RNA polymerase subunit alpha	nucleotide metabolism
YPK_0309	*rplQ*	3.5	large subunit ribosomal protein L17	translation
YPK_0334	*nusG*	1.8	transcriptional antiterminator NusG	transcription
YPK_0338	*rplL*	1.9	50S ribosomal protein L7/L12	translation
YPK_0341	*rpoC*	1.9	DNA-directed RNA polymerase subunit beta'	nucleotide metabolism
YPK_0452	*fis*	3.5	Fis family transcriptional regulator	transcription
YPK_0453		2	tRNA-dihydrouridine synthase B	translation
YPK_0516		4.1	putative anti-sigma B factor antagonist	transcription
YPK_0524*	*rplM*	1.8	large subunit ribosomal protein L13	translation
YPK_0525	*rpsI*	1.9	small subunit ribosomal protein S9	translation
YPK_1066	*rpsB*	2	small subunit ribosomal protein S2	translation
YPK_1681		2.5	hypothetical protein within rpm operon	translation
YPK_1682	*rpmF*	2.5	large subunit ribosomal protein L32	translation
YPK_1822	*rpmI*	2.3	large subunit ribosomal protein L35	translation
YPK_1823	*rplT*	3	large subunit ribosomal protein L20	translation
YPK_2668	*rpsA*	4.6	small subunit ribosomal protein S1	translation
**YPK_2681**	*serS*	1.9	seryl-tRNA synthetase	translation
YPK_3361	*rplS*	2.5	large subunit ribosomal protein L19	translation

YPK_3724	*deaD*	7.5	ATP-dependent RNA helicase	folding, sorting, degradation
YPK_3756	*rpmA*	1.9	large subunit ribosomal protein L27	translation
YPK_3781	*rplI*	1.9	large subunit ribosomal protein L9	translation
YPK_3782	*rpsR*	2.1	small subunit ribosomal protein S18	translation
YPK_3783*	*priB*	2.1	primosomal replication protein B	replication and repair
YPK_4034	*rho*	1.9	transcription termination factor Rho	folding, sorting, degradation
YPK_4185	*dtd*	2	D-tyrosyl-tRNA(Tyr) deacylase	translation
YPK_4186	*rbn*	2.6	ribonuclease BN	RNA processing

Metabolism

Downregulated loci (Crp-activated)

**YPK_0037**	*fdoG*	-2.5	formate dehydrogenase, alpha subunit	energy, C metabolism
**YPK_0364**	*aceB*	-2	malate synthase	C metabolism
**YPK_0365**	*aceA*	-2	isocitrate lyase	C metabolism
**YPK_1001**	*cynT*	-10	carbonic anhydrase	N metabolism
**YPK_1063**	*dapD*	-2.5	2,3,4,5-tetrahydropyridine-2-carboxylate N-succinyltransferase	aa metabolism
**YPK_1131**	*ureA*	-2	urease gamma subunit	nucleotide, aa metabolism
**YPK_1132**	*ureB*	-2	urease beta subunit	nucleotide, aa metabolism
**YPK_1133***	*ureC*	-2	urease alpha subunit	nucleotide, aa metabolism
YPK_1276	*iscS*	-2	cysteine desulfurase	vitamins/cofactor metabolism
YPK_1277	*nifU*	-2	nitrogen fixation protein NifU and related proteins	
YPK_1289	*ndk*	-3.3	nucleoside-diphosphate kinase	nucleotide metabolism
**YPK_1385**	*napF*	-2	ferredoxin-type protein NapF	energy metabolism
YPK_1386*	*napD*	-1.7	periplasmic nitrate reductase	energy metabolism
YPK_1492	*phnA*	-2.5	phosphonoacetate hydrolase	aa metabolism
YPK_1508*	*fadI*	-2	acetyl CoA acyltransferase	lipid metabolism
YPK_1509	*fadJ*	-1.7	3-hydroxyacyl-CoA dehydrogenase / enoyl-CoA hydratase / 3-hydroxybutyryl-CoA epimerase	C, lipid, aa metabolism
YPK_1918	*ldhA*	-2	D-lactate dehydrogenase	C metabolism
YPK_2107	*dadA*	-5	D-amino-acid dehydrogenase	Energy, N, aa metabolism
YPK_2125	*fadD*	-10	long-chain acyl-CoA synthetase	lipid metabolism
YPK_2451	*sdaA*	-2	L-serine dehydratase	aa metabolism
**YPK_2778**	*uxuA*	-2	mannonate dehydratase	C metabolism
**YPK_2784**	*spr*	-5	lipoprotein Spr	metabolism
**YPK_2965**	*sucD*	-2	succinyl-CoA synthetase alpha subunit	C metabolism
**YPK_2966**	*sucC*	-2	succinyl-CoA synthetase beta subunit	C metabolism
**YPK_2967**	*sucB*	-2.5	2-oxoglutarate dehydrogenase E2 component (dihydrolipoamide succinyltransferase)	C, aa metabolism
**YPK_2968**	*kgd, sucA*	-2.5	2-oxoglutarate dehydrogenase E1 component	C, aa metabolism
**YPK_2969**	*sdhB*	-3.3	succinate dehydrogenase iron-sulfur protein	C metabolism
**YPK_2970**	*sdhA*	-3.3	succinate dehydrogenase flavoprotein subunit	C metabolism
**YPK_2971**	*sdhD*	-3.3	succinate dehydrogenase hydrophobic membrane anchor protein	C metabolism
**YPK_2972**	*sdhC*	-3.3	succinate dehydrogenase cytochrome b-556 subunit	energy, C metabolism
YPK_3040	*iolE*	-2	inosose dehydratase	C metabolism
YPK_3041*	*iolC*	-1.7	5-dehydro-2-deoxygluconokinase [	C metabolism
YPK_3046	*idhA; iolG*	-2.5	myo-inositol 2-dehydrogenase	C metabolism
YPK_3048	*iolD*	-2.5	3D-(3,5/4)-trihydroxycyclohexane-1,2-dione hydrolase	C metabolism
YPK_3049	*iolA; mmsA*	-2.5	methylmalonate-semialdehyde dehydrogenase	C, aa metabolism
YPK_3178	*manB*	-1.7	phosphomannomutase	C metabolism
YPK_3228	*ybaW*	-3.3	thioesterase III	lipid metabolism
YPK_3241	*cyoA*	-2.5	cytochrome o ubiquinol oxidase subunit II	energy metabolism
YPK_3242	*cyoB*	-2.5	cytochrome o ubiquinol oxidase subunit I	energy metabolism
YPK_3279	*yajF; mak*	-2	fructokinase	C metabolism
**YPK_3761**	*mdh*	-3.3	malate dehydrogenase	C metabolism
**YPK_3813**	*frdA*	-2	fumarate reductase flavoprotein subunit	C metabolism
**YPK_3814**	*frdB*	-2.5	fumarate reductase iron-sulfur protein	C metabolism
**YPK_3815**	*frdC*	-2.5	fumarate reductase subunit C	C metabolism
**YPK_3816**	*frdD*	-2.5	fumarate reductase subunit D	C metabolism
YPK_3825	*aspA*	-5	aspartate ammonia-lyase	Energy, N, aa metabolism
**YPK_3921**	*rcs*	-3.3	acetyl-CoA synthetase	lipid, C metabolism
YPK_3933	*fadB*	-3.3	3-hydroxyacyl-CoA dehydrogenase / enoyl-CoA hydratase / 3-hydroxybutyryl-CoA epimerase / enoyl-CoA isomerase	lipid, C, aa metabolism
YPK_3934	*fadA*	-3.3	acetyl-CoA acyltransferase	lipid, aa metabolism
**YPK_3950**	*udp*	-2	uridine phosphorylase	nucleotide metabolism
YPK_4144*	*tdh*	-2	threonine 3-dehydrogenase	aa metabolism
YPK_4145	*kbl*	-2.5	glycine C-acetyltransferase	aa metabolism
**YPK_4209**	*rbsK*	-5	ribokinase	C metabolism, PPP
**YPK_4210**	*rbsD*	-5	D-ribose pyranase	C metabolism, PPP

YPK_4219	*atpI*	-2	ATP synthase protein I	energy metabolism
YPK_4221	*atpE*	-2	F-type H+-transporting ATPase subunit C	energy metabolism
YPK_4222*	*atpF*	-2	F-type H+-transporting ATPase subunit B	energy metabolism
Upregulated loci (Crp-repressed)				
YPK_0077	*hutH*	2.8	histidine ammonia-lyase	energy, aa metabolism
YPK_1077	*fabZ*	2.0	Hydroxylmyristoyl-ACP dehydratase	Lipid metabolism
YPK_0150*	*glgA*	3.6	starch phosphorylase	C metabolism
YPK_0151	*glgP*	3.6	starch phosphorylase	C metabolism
YPK_0174	*pckA*	2.1	phosphoenolpyruvate carboxykinase (ATP)	C metabolism
YPK_0494	*treC*	3.5	trehalose-6-phosphate hydrolase	C metabolism
YPK_0495*	*treB*	3.4	trehalose-specific PTS system components IIBC	C metabolism
YPK_0518	*murA*	2	UDP-N-acetylglucosamine 1-carboxyvinyltransferase	C metabolism
YPK_0649	*ribB*	1.9	3,4-dihydroxy 2-butanone 4-phosphate synthase	vitamins/cofactor metabolism
YPK_0663	*plsC*	2.6	1-acyl-sn-glycerol-3-phosphate acyltransferase	lipid metabolism
YPK_1181	*nadB*	2	L-aspartate oxidase	aa metabolism
YPK_1293	*ispG*	2.1	(E)-4-hydroxy-3-methylbut-2-enyl-diphosphate synthase	terpenoids/polyketides metabolism
YPK_1429	*cysK*	3.1	cysteine synthase A	energy, aa metabolism
YPK_1665	*htrB*	2.1	lipid A biosynthesis lauroyl acyltransferase	metabolism, LPS
YPK_1686	*fabG*	3.2	3-oxoacyl-[acyl-carrier protein] reductase	lipid metabolism
YPK_1855	*pykF*	2.8	pyruvate kinase	C, nucleotide metabolism
YPK_2222	*hutI*	1.9	imidazolonepropionase	aa metabolism
YPK_2438	*ftnA*	2.3	ferritin	vitamins/cofactor metabolism
**YPK_2669**	*cmk*	3.3	cytidylate kinase	nucleotide metabolism
**YPK_2867**	*ddc*	3.9	aromatic-L-amino-acid decarboxylase	aa metabolism
YPK_3269	*ggt*	2.8	gamma-glutamyltranspeptidase	lipid metabolism
YPK_3271	*malZ*	2.3	alpha-glucosidase	C metabolism
YPK_3369	*gshA*	2.8	glutamate--cysteine ligase	aa metabolism
YPK_3434	*cysC*	1.8	adenylylsulfate kinase	energy, nucleotide metabolism
YPK_3435	*cysN*	2.4	sulfate adenylyltransferase subunit 1	energy, nucleotide metabolism
YPK_3436	*cysD*	2.5	sulfate adenylyltransferase subunit 2	energy, nucleotide metabolism
YPK_3437	*cysG*	2	uroporphyrin-III C-methyltransferase / precorrin-2 dehydrogenase / sirohydrochlorin ferrochelatase	vitamins/cofactor metabolism
YPK_3440	*cysH*	3.1	phosphoadenosine phosphosulfate reductase	energy metabolism
YPK_3441	*cysI*	3.4	sulfite reductase (NADPH) hemoprotein beta-component	energy metabolism
YPK_3442	*cysJ*	2.4	sulfite reductase (NADPH) flavoprotein alpha-component	energy metabolism
YPK_3446	*eno*	2	enolase	C metabolism
YPK_3449	*relA*	1.8	GTP pyrophosphokinase	nucleotide metabolism
YPK_3863	*ubiA*	2	4-hydroxybenzoate octaprenyltransferase	vitamins/cofactor metabolism
**YPK_3884**	*metF*	4.4	methylenetetrahydrofolate reductase	energy metabolism
YPK_4015	*cyaA*	2.9	adenylate cyclase, class 1	nucleotide metabolism
YPK_4176	*gmk*	1.9	guanylate kinase	nucleotide metabolism
YPK_4229	*glmS*	2	glucosamine--fructose-6-phosphate aminotransferase (isomerizing)	C, aa metabolism
**Transport**				
Downregulated loci (Crp-activated)				
YPK_0431		-3.3	simple sugar transport system substrate-binding protein	transport
**YPK_0558**	*sstT*	-5	serine/threonine transporter	transport
YPK_1144	*celA*	-2.5	PTS system, cellobiose-specific IIB component	transport, PTS
**YPK_1375**	*afuA*	-5	iron(III) transport system substrate-binding protein	transport
**YPK_1376***	*afuB*	-2.5	iron(III) transport system permease protein	transport
**YPK_1377**	*potA*	-2.5	ABC transporter related	transport
YPK_1506	*fadL*	-5	long-chain fatty acid transport protein	transport
**YPK_1538**	*hisJ*	-2.5	histidine transport system substrate-binding protein	transport
**YPK_2067**	*oppD*	-1.7	peptide/nickel transport system ATP-binding protein	transport
**YPK_2068***	*oppC*	-2	peptide/nickel transport system permease protein	transport
**YPK_2069**	*oppB*	-2	oligopeptide transporter permease	transport
**YPK_2070**	*oppA*	-5	peptide/nickel transport system substrate-binding protein	transport
YPK_2464	*manX*	-2	PTS system, mannose-specific IIB component	C metabolism, PTS
YPK_2466	*manZ*	-2	PTS system, mannose-specific IID component	C metabolism, PTS

YPK_2512		-2	major facilitator transporter	transport
YPK_2564	*mglC*	-1.7	methyl-galactoside transport system permease protein	transport
YPK_2565	*mglA*	-2	methyl-galactoside transport system ATP-binding protein	transport
**YPK_2566**	*mglB*	-5	methyl-galactoside transport system substrate-binding protein	transport
YPK_2649	*ompC2*	-5	putative porin	transport
**YPK_2762**	*fruA*	-2.5	PTS system, fructose-specific IIB component, PTS system, fructose-specific IIC component	transport, C metabolism, PTS
**YPK_2763**	*fruK*	-2	1-phosphofructokinase	transport, C metabolism
**YPK_2764**	*fruB*	-2	PTS system, fructose-specific IIA component, phosphocarrier protein FPr	transport, C metabolism, PTS
YPK_2996	*nagE*	-2.5	PTS system, N-acetylglucosamine-specific IIA component, PTS system, N-acetylglucosamine-specific IIB component, PTS system, N-acetylglucosamine-specific IIC component	C metabolism, PTS
**YPK_3010**	*ybeJ, gltI*	-10	glutamate/aspartate transport system substrate-binding protein	transport
**YPK_3011**	*gltJ*	-2.5	glutamate/aspartate transport system permease protein	transport
**YPK_3012***	*gltK*	-2	glutamate/aspartate transport system permease protein	transport
**YPK_3013**	*gltL*	-2	glutamate/aspartate transport system ATP-binding protein	transport
YPK_3044	*rbsA*	-2	ribose transport system ATP-binding protein	transport
YPK_3045	*rbsB*	-2.5	ribose transport system substrate-binding protein	transport
YPK_3398		-1.7	simple sugar transport system substrate-binding protein	transport
YPK_3706		-1.7	multiple sugar transport system ATP-binding protein	transport
**YPK_3707***		-2	multiple sugar transport system permease protein	transport
YPK_3709		-2	extracellular solute-binding protein	transport
**YPK_3923**	*actP*	-3.3	cation/acetate symporter, permease	transport
Upregulated loci (Crp-repressed)				
YPK_0495	*treB*	3.2	PTS system, trehalose-specific IIB component, PTS system, trehalose-specific IIC component	C metabolism, PTS
YPK_0512	*yrbF*	2.9	putative ABC transport system ATP-binding protein	transport
YPK_0513	*yrbE*	3	putative ABC transport system permease protein	transport
YPK_0514	*yrbD*	2.6	putative ABC transport system substrate-binding protein	transport
YPK_0698	*fepB*	2.6	iron complex transport system substrate-binding protein	transport
YPK_1408	*cysP*	2.3	sulfate transport system substrate-binding protein	transport
YPK_1409	*cysU*	2.3	sulfate transport system permease protein	transport
YPK_1410	*cysW*	2	sulfate transport system permease protein	transport
YPK_1411	*cysA*	3.3	sulfate transport system ATP-binding protein	transport
YPK_1438	*nupC1*	2.9	nucleoside transport protein	transport
**YPK_2752**	*lysP*	6.5	lysine-specific permease	transport
**YPK_2839**	*ompC*	2	outer membrane pore protein C	transport
**General membrane transport, secretion, and structural proteins**				
Downregulated loci (Crp-activated)				
YPK_0736	*slyB; pcp*	-2.5	outer membrane lipoprotein	membrane and intracellular structural molecules
**YPK_3923**	*actP*	-3.3	cation/acetate symporter, permease	transporter
YPK_3942	*tatA*	-1.7	sec-independent protein translocase protein TatA	protein export
Upregulated loci (Crp-repressed)				
YPK_0025	*yiaF*	5.7	putative lipoprotein	membrane and intracellular structural molecules
YPK_0257		1.8	lysine exporter protein LysE/YggA	transporter
YPK_0303	*secY*	2.8	preprotein translocase subunit SecY	protein export
YPK_0333	*secE*	1.8	preprotein translocase subunit SecE	protein export
**YPK_2956**	*tolB*	1.8	translocation protein TolB	cell motility, secretion
YPK_0465	*mreB*	2.2	rod shape-determining protein	cell motility, cytoskeleton
YPK_1074	*yaeT*	2.1	outer membrane protein	membrane and intracellular structural molecules
YPK_1844		2.4	putative inner membrane protein	membrane and intracellular structural molecules
YPK_2549	*terC*	2.4	integral membrane protein TerC	membrane and intracellular structural molecules
**YPK_3028**	*tatE*	2.4	sec-independent protein translocase protein TatE	protein export
YPK_3216	*ybaY*	1.9	putative lipoprotein	membrane and intracellular structural molecules
**Others**				
Downregulated loci (Crp-activated)				
pYptb0001	*rep*	-3.3	plasmid replication protein	
pYV0007		-2.5	replication protein	

pYV0017		-1.7	putative resolvase	
YPK_0446		-1.7	beta-lactamase domain-containing protein	
YPK_0563	*fadH*	-2	uncharacterized 2,4-dienoyl-CoA reductase (NADPH2)	
YPK_0651		-2	glutathionylspermidine synthase	
YPK_1878	*anmK*	-2.5	anhydro-N-acetylmuramic acid kinase	
YPK_1976	*ydfG*	-2.5	3-hydroxy acid dehydrogenase	
YPK_1981	*mlc*	-2	ROK family protein	
YPK_2095	*msrB*	-2	peptide-methionine (R)-S-oxide reductase	
YPK_2437	*copC*	-1.7	copper resistance protein CopC	
YPK_2502		-2	polysaccharide deacetylase	
**YPK_2508**		-2.5	mandelate racemase/muconate lactonizing protein	
**YPK_2510**	*ucpA*	-2	short-chain dehydrogenase/reductase SDR	
YPK_3187		-2	glycosyl transferase family protein	
YPK_3205	*ychN*	-2	uncharacterized protein involved in oxidation of intracellular sulfur	
YPK_3301*	*nqrE*	-2	Na+-transporting NADH:ubiquinone oxidoreductase subunit E	
YPK_3303	*nqrC*	-2	Na+-transporting NADH:ubiquinone oxidoreductase subunit C	
YPK_3304*	*nqrB*	-2	Na+-transporting NADH:ubiquinone oxidoreductase subunit B	
YPK_3305	*nqrA*	-2.5	Na+-transporting NADH:ubiquinone oxidoreductase subunit A	
**YPK_3788**	*aidB*	-2.5	putative acyl-CoA dehydrogenase	
Upregulated loci (Crp-repressed)				
YPK_0515	*yrbC*	3.8	putative toluene tolerance protein	
**YPK_0789**		3.7	intradiol ring-cleavage dioxygenase	
YPK_1544	*yfcE*	2.1	phosphodiesterase	
YPK_1545	*yfcD*	2.1	NUDIX hydrolase	
**YPK_1760**	*ampD*	1.9	N-acetylmuramoyl-L-alanine amidase	
YPK_1778		2	spore coat U domain-containing protein	
YPK_1811		2.7	fructosamine kinase	
YPK_1903	*tpx*	1.9	thiol peroxidase, atypical 2-Cys peroxiredoxin	
YPK_2260		1.8	amidohydrolase 2	
**YPK_2470**	*atoS1*	2.9	diguanylate cyclase with PAS/PAC sensor	
YPK_2488		2.2	RIO kinase 1	Metabolism; Enzyme Families; Protein kinases
**YPK_2746**		1.8	HAD family hydrolase	
**YPK_2836**	*ampH*	2.1	beta-lactam binding protein AmpH	
**YPK_2854**		3.2	YfaZ family protein	
**YPK_2954**	*ybgF*	2.1	tol-pal system protein YbgF	
**YPK_3120**		1.9	putative bacteriophage protein	
**YPK_3122**		2.1	Mu tail sheath family protein	
YPK_3362	*trmD*	1.9	tRNA (guanine-N1-)-methyltransferase	
YPK_3371	*yqaB*	3.6	fructose-1-phosphatase	
YPK_3505		2.3	hypothetical protein	
**YPK_3607**	*creA*	4.6	CreA protein	
YPK_3775	*cysQ*	2.2	adenosine-3'(2'),5'-bisphosphate nucleotidase	
YPK_4184		1.9	thioesterase domain-containing protein	
**YPK_4187**		5.6	putative hydrolase of the HAD superfamily	
**Hypothetical**				
Downregulated loci (Crp-activated)				
pYptb0010		-2	hypothetical protein	
YPK_0598		-2	hypothetical protein	
**YPK_0631**		-3.3	hypothetical protein	
YPK_0652		-2	hypothetical protein	
**YPK_1062**		-1.7	hypothetical protein	
YPK_1322		-2	hypothetical protein	
**YPK_1326**		-2	hypothetical protein	
YPK_1510		-2	hypothetical protein	
YPK_1601		-3.3	hypothetical protein	
YPK_1725		-2	hypothetical protein	
YPK_1818		-5	hypothetical protein	
YPK_1842		-2	hypothetical protein	
YPK_1989		-10	hypothetical protein	
YPK_1990		-10	hypothetical protein	
**YPK_2018**		-3.3	hypothetical protein	
YPK_2059		-2	hypothetical protein	
YPK_2094		-2	hypothetical protein	
**YPK_2185**		-10	hypothetical protein	
YPK_2219		-2.5	hypothetical protein	
YPK_2294		-2	hypothetical protein	

YPK_2295		-2	hypothetical protein
YPK_2475		-3.3	hypothetical protein
**YPK_2483**		-10	hypothetical protein
YPK_2663		-2	hypothetical protein
**YPK_2804**		-2	hypothetical protein
**YPK_3035**		-10	hypothetical protein
YPK_3203		-2.5	hypothetical protein
YPK_3240		-2	hypothetical protein
**YPK_3281**	*yaiE*	-5	hypothetical protein
YPK_3285		-1.7	hypothetical protein
YPK_3298		-2.5	hypothetical protein
**YPK_3922**		-5	hypothetical protein
YPK_4208		-2.5	hypothetical protein
Upregulated loci (Crp-repressed)			
**YPK_0497**		11	hypothetical protein
**YPK_0536**		2	hypothetical protein
**YPK_1762**		2.5	hypothetical protein
**YPK_1951**		3.7	hypothetical protein
**YPK_2197**		2.1	hypothetical protein
**YPK_2198**		2.2	hypothetical protein
**YPK_2199**		2.4	hypothetical protein
YPK_2637		1.8	hypothetical protein
**YPK_2868**		4.8	hypothetical protein
**YPK_3123**		2	hypothetical protein
YPK_3370	*yqaA*	3.1	hypothetical protein
**YPK_3567**		9.6	hypothetical protein
**YPK_3608**		3.5	hypothetical protein
YPK_3763		5.4	hypothetical protein
YPK_3879		2	hypothetical protein
**YPK_3880***		2.2	hypothetical protein
**YPK_3882***		2	hypothetical protein
**YPK_4107**		8	hypothetical protein
**YPK_4108**		4.2	hypothetical protein
YPK_4111		2.2	hypothetical protein

The relative expression (fold change) of these genes in the wildtype and the *crp* mutant is given for bacteria grown at 25°C in LB. Genes of the different categories were selected according to the KEGG database or identified by BLAST searches with functionally characterized proteins from *Y. pseudotuberculosis* and/or *Y. pestis* strains. Significantly increased or decreased transcript levels (change >1.8 fold) are shown. Genes which are also differentially regulated in a *csrA* mutant strains are given in bold. * indicates genes which have been identified with only one RNA probe, all other have been identified with two or three RNA probes.

Table S3. qRT-PCR of Crp target transcripts.

Gene ID	Gene locus	Fold change Microarray	Fold change RT PCR	p value RT PCR
YPK_1606	*ompX; ailD*	6.9	65.1	< 0.0001
YPK_1818		-5	-10.1	0.0234
YPK_2070	*oppA*	-5	-2.3	0.0024
YPK_2107	*dadA*	-5	-3.8	0.0010
YPK_2566	*mglB*	-5	-12	0.0358
YPK_2996	*nagE*	-2.5	-1.5	0.0089
YPK_3010	*ybeJ; gltI*	-10	-10.6	0.0002
YPK_3035		-10	-18.1	0.0028
YPK_3724	*deaD*	7.5	30.2	0.0015
YPK_3950	*udp*	-2	-16.7	0.0005

Six independent cultures of the *Y. pseudotuberculosis* wildtype strain YPIII and the isogenic *crp* mutant strain YP89 were grown in LB medium overnight at 25°C and total RNA was prepared. The total RNA of two individual RNA preparations was pooled and used for DNase digestion. qRT-PCR was performed with three biological triplicates and two technical replicates. Gene expression levels were normalized to levels of the 5S rRNA transcript according to Pfaffl *et al.* 2001 and are given as relative values Δ*crp*/wildtype.

Table S4. Bacterial strains and plasmids.

Strains, Plasmids	*Description*	*Source and reference*
Bacterial strains		
E. coli		
BL21λDE3	F^- *ompT gal dcm lon hsdSB* (r_B^- m_B^-) λ DE3	(Studier and Moffatt, 1986)
CC118λpir	F^- Δ(*ara-leu*)7697 Δ(*lacZ*)74 Δ(*phoA*)20 *araD139* *galE galK thi rpsE rpoB arfE*^{am} *recA1*, *λpir*	(Manoil and Beckwith, 1986)
S17-λpir	*recA1 thi pro hsdR*$^-$ RP4-2Tc::Mu Km::Tn7 λpir	(Herrero et al., 1990)
Y. pseudotuberculosis		
YPIII	pIB1, wildtype	(Bolin et al., 1982)
YP3	pIB1, *rovA*::Tn*10*(60)[a]; Cm^R	(Nagel et al., 2001)
YP48	pIB1, Δ*csrC*, Kn^R	(Heroven et al., 2008)
YP53	pIB1, Δ*csrA*, Kn^R	(Heroven et al., 2008)
YP69	pIB1, Δ*csrB*, Ap^R	(Heroven et al., 2008)
YP72	pIB1, Δ*rovM*, Kn^R	this study
YP79	pIB1, Δ*csrB*, Δ*csrC*	this study
YP80	pIB1, Δ*hfq*	this study
YP87	pIB1, Δ*uvrY*, Kn^R	this study
YP88	pIB1, Δ*crp*, Kn^R	this study
YP89	pIB1, Δ*crp*	this study
YP106	pIB1, Δ*csrC*, Kn^R	this study
YP107	pIB1, Δ*rovA*	(Quade et al., 2012)
YP120	pIB1, Δ*uvrY*	this study
YP124	pIB1, Δ*crp*, Δ*csrC*, Kn^R	this study
YP125	pIB1, Δ*crp*, Δ*uvrY*, Kn^R	this study
YP126	pIB1, Δ*csrC*	this study
YP127	pIB1, Δ*crp*, Δ*csrC*	this study
YP128	pIB1, Δ*crp*, Δ*uvrY*	this study
Plasmids		
pACYC184	cloning vector, p15A, Cm^R, Tet^R	(Chang and Cohen, 1978)
pAKH3	pGP704, *sacB*$^+$, Ap^R	this study

pAKH37	pACYC184, *crp*$^+$, Cm^R	this study
pAKH47	pGP20, *rovA'-'lacZ*(17)[a], Tet^R	(Heroven and Dersch, 2006)
pAKH52	pACYC184, *csrC*$^+$, Cm^R	(Heroven et al., 2008)
pAKH58	pGP20, *flhDC´-´lacZ*(26)[a], Tet^R	(Heroven et al., 2008)
pAKH59	pACYC184, *csrC*$^+$, Cm^R	(Heroven et al., 2008)
pAKH63	pGP20, *rovM'-'lacZ*(41)[a], Tet^R	(Heroven and Dersch, 2006)
pAKH75	pACYC184, *uvrY*$^+$, Cm^R	(Heroven et al., 2008)
pAKH85	pACYC184, Cm^R, Tet^S	(Heroven and Dersch, 2006)
pAKH101	pHT124, *csrB-lacZ*(4)[b], Ap^R	(Heroven et al., 2008)
pAKH105	pGP20, *hfq'-'lacZ*(3)[a], Tet^R	this study
pAKH125	pHT124, *csrC-lacZ*(4)[b], Ap^R	this study
pAKH149	pAKH3, *csrC*::Kan^R	this study
pAKH151	pAKH3, *uvrY*::Kan^R	this study
pAKH171	pET28a, *crp*$^+$, Kan^R	this study
pAY01	pGP81 *sacB*, Ap^R	(Yang and Isberg, 1993)
pET28a	T7 overexpression vector, Kan^R	Novagen
pFU98	promoter probe vector, *rbs-luxCDABE*, Cm^R	(Uliczka et al., 2011)
pGP20	protein fusion vector, pSC101, *´lacZ*, Tet^R	Petra Gerlach
pGP704	R6K cloning vector	(Miller and Falkow, 1988)
pHT124	promoter probe vector, *lacZ*$^+$, Ap^R	(Heroven et al., 2008)
pKB6	pGP20, *barA-lacZ*(2)[a], Tet^R	this study
pKB7	pGP20, *uvrY-lacZ*(3)[a], Tet^R	this study
pKD4	kanamycin cassette template, Kan^R, Ap^R	(Datsenko and Wanner, 2000)
pKD46	recombination vector, λ RED recombinase, Ap^R	(Datsenko and Wanner, 2000)
pWO25	pFU98, *csrB-luxCDABE*(1)[b], Cm^R	this study

[a] The number indicates the codon of the corresponding gene fused to *lacZ*.

[b] The number indicates the nucleotide of the corresponding gene fused to *lacZ* or *luxCDABE*.

Table S5. Oligonucleotides used in this study.

Number	sequence	Site[a]
409	GGGCCCGGATCCCGACATCAATGGCGCTACAC	*Bam*HI
410	GCGGCGGTCGACGCTAATAGCTTCAGCTTTAAC	*Sal*I
I663	CCGGCCGAATTCGTGAAACCTTACCTTACCGG	*Eco*RI
I664	GGGGCCGAATTCCCTTAGCCATTCTATATTTTCC	*Eco*RI
II157	GCGGCGGTCGACCCTTCATCCCGTGGTAGG	*Sal*I
I363	GGGCCGGTCGACCCAATAACAAATTGACTAGC	*Sal*I
I661	GTGTAGGCTGGAGCTGCTTC	
I662	CATATGAATATCCTCCTTAGTTCC	
II850	GCGGCGGAGCTCCACTGATGACGAAGTGAGTC	*Sac*I
II851	**GAAGCAGCTCCAGCCTACAC**CAAATTGACTAGCTGATTGC	
II852	**ACTAAGGAGGATATTCATATG**CTGCTAGATTCCGGCCC	
II853	GCGGCGGAGCTCGGTTCTCGCACCTGAGCG	*Sac*I
II858	GCGGCGGAGCTCCCTCATAGGAATTAACTTATC	*Sac*I
II859	**GAAGCAGCTCCAGCCTACAC**GAAATTTCTCCAGAAATAAGG	
II860	**ACTAAGGAGGATATTCATATG**GGAGACGTTATCAAATAGTG	
II861	GCGGCGGAGCTCCTTATCATCACGTAGCAACA	*Sac*I
I350	CGGGCCTGCAGGCTAATCGCTCAGATGACTG	*Pst*I
I351	CG GGCCTGCAGGTCATGGTATTCCAATGTGGG	*Pst*I
I348	CGGGCCTGCAGCTTTGCTGTATCAATGCTTGC	*Pst*I
I349	CGGGCCTGCAGCTGATCAAAGAAATTTCTCCAG	*Pst*I
III417	GCACTGGATCCGAGTGAGGATGATTTCATCCG	*Bam*HI
III418	GCACTGTCGACAGTAAAGGATCGCTTAATCAG	*Sal*I
IV787	GCGCCGCTAGCGTTCTCGGTAAGCCACAAACAGA	*Nhe*I
IV788	GGCGGCTCGAGGAGGGAATTAACGGGTGCCG	*Xhe*I
636	GAGTGCACTGGATTTACCC	
I659	**GAAGCAGCTCCAGCCTACAC**CAAATTGACTAGCTGATTGC	
I660	**ACTAAGGAGGATATTCATATG**CTGCTAGATTCCGGCCCAATC	
639	CCAGACGTTCGTCATTCTCC	
II235	GACACGACATCAATGGCGC	
II236	**GAAGCAGCTCCAGCCTACAC**CGCTGTTATCCTCTGTTGTTATC	
II237	**ACTAAGGAGGATATTCATATG**GCACCCGTTAATTCCCTC	
II238	CATGCTGAGACTGAAAATGG	
I665	CCAATTTGCGATTGCGCCTG	
I666	**GAAGCAGCTCCAGCCTACAC**CTATATTTTCCTTATTTGCTTGTTG	
I667	**ACTAAGGAGGATATTCATATG**GCCCATTGCTGGTCGACC	
I668	GGTACGAGCTCGTTGGGC	
I63	GATAACGCTGATACTTAAAACACCAGGGTAGTTTGTAATTAGAATTC**G**	

	**TGTAGGCTGGAGCTGCTTC**	
I64	CCTTAAAATACAGGGGCTTGTGATGATGAATTAAAAACAGATTAC**CAT ATGAATATCCTCCTTAGT**	
555	CGGCGCGGATCCCTCTCACACCAGCTGTG	
556	GGGGGCGTCGACGGCAAACTCAATATCCTG	
I82	GCAATCAGCTAGTCAATTTG	
583	GGGCGCGGATCCGATTGGGCCGGAATCTAGC	
IV737	GCATGATGGTTGGCGCATACG	
IV738	CGCCCTTAGCCAGCAATACG	
IV741	GGAGCGACGTTGGCTATCGT	
IV742	GGAAGGTGCGCACGCCAAG	
IV743	GGTGGTTATGCCCAGAGCGA	
IV744	CGGTATGCTGGACCACCGG	
IV749	CGCTGTGTTCGCCAGCAGC	
IV750	GGAACCAAACGACGACCACTG	
IV751	GTGCATTTGTGATATTAGCGTTG	
IV752	CTGGCTGCATTCTCCGCG	
IV753	GGCGTCAAGGTGGTGGCCC	
IV754	CGGGATCTGCGCCATAATCG	
IV761	CACGTGATCTACTTGAAGGGC	
IV762	GCAAATAGCTTGCATAAGGAGAG	
IV765	CCGTTTGGATGGCAGCAGCC	
IV766	CACCGGCATCATCCAATACGG	
IV767	CGGTGTTATCGTTGTGGGCC	
IV768	CCGCTTGCTGCTGACGTTCC	
IV769	GTCAACACTAGAGAAGCTGGTT	
IV770	CAACGGCCTGTAAATATCCCTG	
II812	GCCTGGCGGCCATAGCGC	
II813	GCCTGGCAGTGTCCTACTCT	
837	GGAAGGAAATCGTGACATTC	
II431	CGGCGCGTCGACGATCAAAGAAATTTCTCCAG	
II846	GCGGCGGAGCTCGGCTAAAGCTCTCGCCAAC	
I438	GGGCCGGTCGACCCAGCGAGTAAAGGATCGC	
I362	GCGGCGGAATTCGACACCATCAACCACTTC	
I363	GGGCCGGTCGACCCAATAACAAATTGACTAGC	
IV853	CAGTACCCCTCATCAGGC	
IV854	CAATACGAATGTTATTTGACATG	
185	GCGCGCGTCGACCCAAAACCAAATCTGAAAGC	
147	GCGGCGGGATCCGCGCCAAACGCGAACTAATCG	

The corresponding restriction sites are underlined[a]. Nucleotides homologous to the kanamycin resistance cassette are given in bold.
